# Predictive Analysis of Social Determinants of Health in Posterior Cervical Spine Surgery

**DOI:** 10.3390/jcm15187047

**Published:** 2026-09-11

**Authors:** Mehul Mittal, Rishi Jain, Joshua M. Tennyson, Divy Kumar, Pranav M. Bajaj, Samuel G. Reyes, Wellington K. Hsu, Alpesh A. Patel, Srikanth N. Divi

**Affiliations:** Department of Orthopaedic Surgery, Northwestern University Feinberg School of Medicine, Chicago, IL 60611, USA; rishi.jain@northwestern.edu (R.J.); joshua.tennyson@northwestern.edu (J.M.T.); divy.kumar@northwestern.edu (D.K.); pranav.bajaj@northwestern.edu (P.M.B.); samuel.reyes@northwestern.edu (S.G.R.); wellington.hsu@nm.org (W.K.H.); alpesh.patel@nm.org (A.A.P.); srikanth.divi@nm.org (S.N.D.)

**Keywords:** machine learning, social determinants of health, PCDF, readmission, healthcare utilization

## Abstract

**Background/Objectives:** Posterior cervical decompression and fusion (PCDF) carry perioperative risks and increases postoperative healthcare utilization (HU). Traditional prediction models emphasize comorbidities and surgical factors, yet social determinants of health (SDHs) are known to also affect clinical outcomes. We applied machine learning (ML) to integrate SDHs and clinical variables in predicting 90-day readmission and HU after PCDF. **Methods:** We conducted a retrospective, single-institution machine learning analysis of adult patients undergoing single or multilevel PCDF (2003–2023). Models were designed using 88 clinical variables and five census-derived Social Vulnerability Index (SVI) scores. Outcomes were 90-day readmission and HU (the unweighted sum of 18 post-discharge components including urgent visits, invasive procedures, non-routine testing, and imaging). Models were trained on 50 repeated 80/20 train–test splits with training-only preprocessing and hyperparameter tuning. **Results:** Among 1015 patients (mean age of 64.7, 59.0% male, 97.2% with multilevel fusions), 90-day readmission was 15.5% and mean HU score was 13.6 ± 9.0. Regarding readmission, the clinical-only logistic regression model was the best predictor (AUROC 0.65). For HU, clinical-only random forest performed best (MAE: 4.80, R^2^: 0.326). Adding SVI to the matched clinical-plus-SDH models did not improve prediction for either 90-day readmission or healthcare utilization. Length of stay, year of surgery, and several SVI measures were prominent contributors across both SHAP analyses. **Conclusions**: ML models integrating SDH and clinical factors available by index discharge modestly predicted readmission and HU after PCDF. However, adding SVI did not meaningfully increase their overall performance, and the models require external validation before clinical use.

## 1. Introduction

Posterior cervical decompression and fusion (PCDF) is a widely used surgical approach for the treatment of degenerative cervical spine pathologies, particularly in patients with multilevel spondylotic myelopathy, severe radiculopathy, or cervical deformity inadequately resolved by anterior approaches [1]. Compared to its anterior counterpart (ACDF), PCDF is often performed in older patients with greater comorbidity burden and more extensive fusion constructs. Although not as common, PCDF procedures increased 2.7-fold between 2001 and 2013; despite its effectiveness in symptom alleviation and restoring functional capacity, PCDF is associated with perioperative risk, prolonged recovery, and high rates of postoperative healthcare utilization [1,2].

The optimization of perioperative care is hindered by a poor understanding of how to best identify patients at the greatest risk for adverse outcomes. Traditional prediction models have emphasized patient comorbidities such as diabetes, smoking, and obesity, as well as operative factors including number of levels fused and procedural time. However, social determinants of health (SDHs) are becoming increasingly recognized as a factor in shaping perioperative trajectories [3]. Factors including socioeconomic status, education, access to transportation, and social support—among many others—may contribute to patients’ abilities to access adequate care and engage in health maintenance. Previous work has demonstrated that socioeconomic disadvantage, racial and ethnic minority statuses, and poor baseline access to healthcare resources are associated with increased complication rates and greater likelihoods of readmission [3,4]. However, such work has either taken a macroscopic view of spine surgery collectively or focused on ACDF cohorts; the influence of SDHs on outcomes specifically after PCDF remains poorly defined [4].

Healthcare utilization (HU) is the summation of all post-discharge resources used by a patient in relation to an index procedure, and it represents a pertinent endpoint for PCDF given the degree of postoperative burden for patients. Unlike readmission, which captures a single event, HU reflects a broader range of postoperative needs and better informs us on future health-system planning. Specifically, HU includes emergency department and urgent care visits, reoperations, invasive testing, and non-routine imaging [5]. Understanding how SDH interacts with both clinical and non-clinical attributes of a patient could clarify how social vulnerability affects postoperative recovery and enables more personalized perioperative interventions. Machine-learning (ML)-based approaches provide a valuable tool for such analyses. ML algorithms are well-suited to uncover non-linear and interactive relationships within high-dimensional data. Unsupervised and supervised methods, though only modestly accurate, have been successfully applied to predict outcomes for spine surgery patients [6]. The use of explainable artificial intelligence (XAI) approaches, including Shapley Additive Explanations (SHAPs), also improve the clinical interpretability of these models by estimating and visualizing the contribution of individual variables to final model output without implying causality [7]. To our knowledge, this study is among the first to leverage ML and XAI to fine-tune prediction specifically in the PCDF patient population. The aim of our present study is to apply several ML algorithms to a large, single-institution cohort of PCDF patients. We incorporate over 80 clinical and SDH-oriented variables to evaluate whether census-tract SDH measures provide incremental predictive value beyond clinical variables for 90-day readmission and postoperative HU after PCDF.

## 2. Materials and Methods

### 2.1. Patient Cohort and Study Variables

Adult patients who underwent single or multilevel PCDF for degenerative cervical disease at our single integrated academic health system, including the primary hospital and affiliated satellite sites, between 2003 and 2023 were identified from the electronic data warehouse (EDW). Patients were excluded if the surgical indication was malignancy, trauma, or primary spinal infection, which resulted in a total of 1050 patients identified. Patients were then additionally excluded if analysis data were incomplete, yielding a final analytic cohort of 1015 patients. No imputation was performed.

The prediction time point was defined as the end of index hospitalization. A total of 88 clinical variables and five SDH variables, including demographics, past medical history, past surgical history, preoperative medication/drug exposure, comorbidity and frailty measures, and total preoperative duration were included as key predictors for readmission and HU (Table 1). Readmissions included any admission to a hospital within 90 days of discharge, including admissions for systemic medical complications, persistent cervical spine symptoms, or complications directly attributable to PCDF. Readmissions were ascertained from the institutional electronic medical record (EMR), including any outside-hospital encounters that were available in the EMR through partnerships with local hospital systems. HU was evaluated as a continuous secondary outcome and calculated as the unweighted sum of 18 post-discharge components within 90 days (Table 1). Post-discharge medications, postoperative complications, readmission indicators, and HU components were excluded to prevent temporal leakage and circular prediction. The clinical-only feature set comprised 88 candidate predictors. The clinical-plus-SDH feature set contained these 88 predictors plus five census-tract SVI measures for 93 predictors in total. A complete inventory and coding description of all candidate predictors is provided in Supplementary Table S7.

To obtain SDH data, each patient’s home address at the time of surgery was abstracted from the EMR. The Geocodio platform (geocod.io) was then used to map addresses to census tracts, and census tracts were linked to CDC/ATSDR SVI releases temporally matched to surgery year. Specifically, patients were matched to 2010 SVI data for surgery years 2012 and earlier, 2014 SVI data for 2013–2014, 2016 SVI data for 2015–2016, 2018 SVI data for 2017–2018, and 2020 SVI data for 2019 onward [8]. SVI, a census-based metric which describes the summative effects of a collective of SDH factors, is categorized into four primary themes: socioeconomic status, household characteristics, racial and ethnic minority statuses, and housing type and transportation [9]. Per the SVI, the metric of household characteristics is measured by these factors: age ≥ 65 years, age ≤ 17 years, disability, single-parent households, and limited English proficiency [6]. Each sub-domain is scored from 0 to 1 at the census tract level, with higher scores reflecting greater social vulnerability. All the sub-domain scores are summed to produce an overall SVI rank.

### 2.2. Machine Learning Approaches

ML models were implemented in Python (version 3.13.15), and performance was validated by area under the curve (AUROC) for prediction of 90-day readmission and mean absolute error (MAE) for prediction of HU. Readmission classification included logistic regression, elastic-net logistic regression, balanced random forest, XGBoost, neural network, and prevalence-only models. HU regression models included support vector regression, random forest, and XGBoost. Each model was fit separately using the clinical-only and clinical-plus-SDH feature sets to assess the incremental predictive value of adding SVI measures. Continuous variables were standardized while binary variables were passed through unchanged.

For each outcome, 50 repeated 80/20 training/testing sets were performed. Classification splits were stratified by readmission status and regression splits by HU quintile. A Bayesian Search Algorithm with over 25 iterations and across inner 5-fold cross-validation was used for hyperparameter optimization. The best parameters selected from hyperparameter optimization were selected based on the mean AUROC for classification and negative MAE for regression models. Test data were not used to fit preprocessing, hyperparameters, thresholding, or recalibration models. Balanced class weights and scale-pos-weight adjustment (XGBoost) were used to address class imbalance. Classification thresholds were selected by maximizing F1 using out-of-fold training predictions, which were then applied to test data.

Classification model evaluation included AUROC, AUPRC, Brier score, expected calibration error, calibration slope and calibration intercept, specificity, sensitivity, positive predictive value (PPV), negative predictive value (NPV), F1 score, and accuracy. HU regression performance was evaluated using MAE, RMSE, R^2^, and median absolute error. Performance metrics were summarized as mean ± SD across the 50 repetitions and as means with empirical 2.5th and 97.5th percentile intervals in Table 2. The best-performing models were determined by the highest mean AUROC for readmission and lowest mean MAE for HU. SHAP values were calculated for the best-performing clinical-plus-SDH model for each outcome. Because class-weighted classification models showed overprediction of absolute readmission risk, we performed a post hoc nested recalibration sensitivity analysis using Platt scaling and isotonic calibration fit on out-of-fold training predictions and applied to held-out test predictions. Sensitivity analyses included deduplicated predictor sets, log-transformed HU, parsimonious clinical baseline models, restricted SVI-theme models, principal component analysis-based clinical reduction, and post hoc recalibration assessment. Decision-curve analysis was performed for the 90-day readmission models to estimate net benefit across a range of clinically plausible risk thresholds; threshold values were evaluated across the predicted-probability range used in the repeated test splits. For Table 3, baseline characteristics were compared using Welch’s t-test for continuous variables and chi-square or Fisher’s exact tests for categorical variables, as appropriate. For paired model comparisons, matched clinical-only and clinical-plus-SDH models were evaluated on identical repeated train–test splits so that performance differences reflected feature-set changes rather than split variability. For HU regression, the dummy comparator predicted the median HU value from the training data. Post hoc Platt recalibration was performed for readmission models by fitting a logistic recalibration model on out-of-fold training predictions and applying the recalibration model to held-out test predictions. All ML models were run using their respective implementations in Python: scikit-learn, shap, Tensorflow, and Keras packages.

Additional post hoc sensitivity analyses assessed domain-specific HU definitions and temporal model validity. HU components were grouped into four clinically defined unweighted domain sums: outpatient visits, acute care utilization, referrals, and imaging/procedures, with the total HU score retained as the primary reference outcome (Supplementary Table S14). Temporal validation was performed by training models from earlier surgery years and evaluation of models from later years used three prespecified cutoffs: training through 2017 with testing in 2018 or later; training through 2018 with testing in 2019 or later; and training through 2019 with testing in 2020 or later. The training-through-2018/testing-in-2019-or-later split was designated as the primary temporal analysis, and hyperparameter tuning was restricted to the training period.

## 3. Results

A total of 1015 patients met the inclusion criteria and were included in our analysis (Table 4). The average age of the patients included was 64.7 years (SD of 11.2). Our cohort had a greater number of males (59.0%), multilevel fusions (97.2%), and ASA class of three or greater (74.1%). The 30-day and 90-day readmission rates were 10.9% and 15.5%, respectively. The mean HU score was 13.6 ± 9.0, with a median of 12, an IQR of 9 to 15, and a range of 1 to 98. When stratified by 90-day readmission status and HU above or below the cohort median, patients with readmission or above-median HU had longer index lengths of stay and greater perioperative burden, while SVI percentile distributions were similar across groups [Table 3].

The clinical-only logistic regression model performed the best for prediction of 90-day readmission with an AUROC of 0.646 ± 0.045, AUPRC of 0.278 ± 0.048, and Brier score of 0.226 ± 0.009 [Table 2]. The clinical-plus-SDH logistic regression model had a mean AUROC of 0.635 ± 0.043, AUPRC of 0.270 ± 0.048, and Brier score of 0.227 ± 0.009. Before recalibration, the class-weighted readmission models overpredicted absolute risk. In the clinical-plus-SDH logistic regression model, Platt recalibration improved the Brier score from 0.227 to 0.126 and expected calibration error from 0.313 to 0.033. Mean predicted risk decreased from 0.464 to 0.156, approximating the observed readmission prevalence of 0.153. At the training-derived threshold, the clinical-only logistic regression model had a sensitivity of 0.466 and specificity of 0.752. Decision-curve analysis did not indicate a positive net benefit across clinically relevant thresholds. Additional calibration measures, threshold-dependent metrics, and confusion-matrix counts are reported in the Supplementary Data.

The clinical-only random forest model performed best overall for prediction of HU with an MAE of 4.797 ± 0.320, RMSE of 7.369 ± 0.834, and R^2^ of 0.326 ± 0.111 (Table 2). Within the clinical-plus-SDH feature set, SVR performed best, with a mean MAE of 4.838 ± 0.290, RMSE of 7.846 ± 0.905, and R^2^ of 0.244 ± 0.053. Across matched model families, adding SVI did not improve held-out performance for either 90-day readmission or HU.

**Figure 1:**
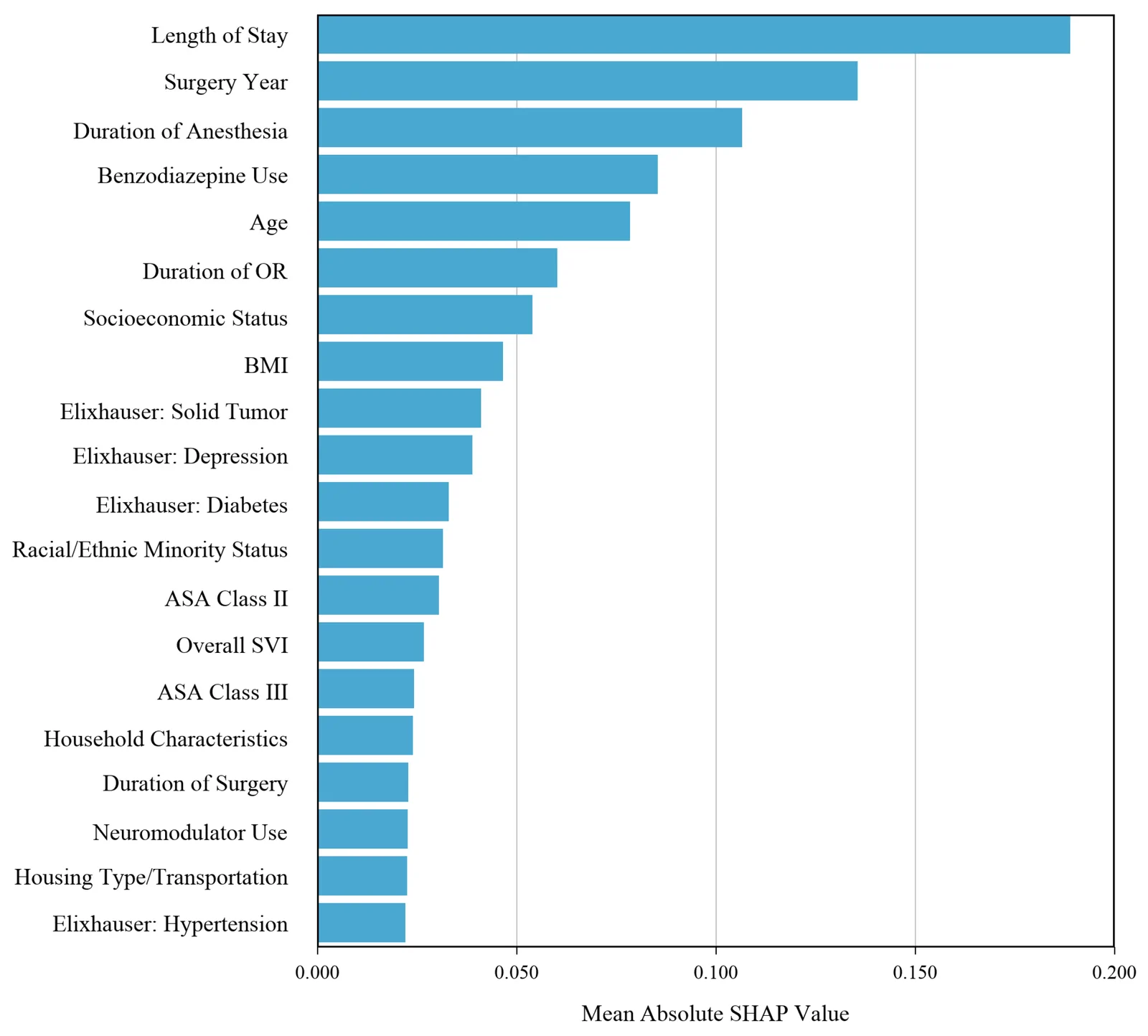
SHAP feature importance for 90-day readmission prediction. Feature importance plot for the clinical-plus-SDH logistic regression model predicting 90-day post-discharge readmission. The x-axis represents the mean absolute SHAP value, calculated as the average absolute contribution of each predictor to the model output. Larger values indicate greater average model attribution to model predictions.

**Figure 2:**
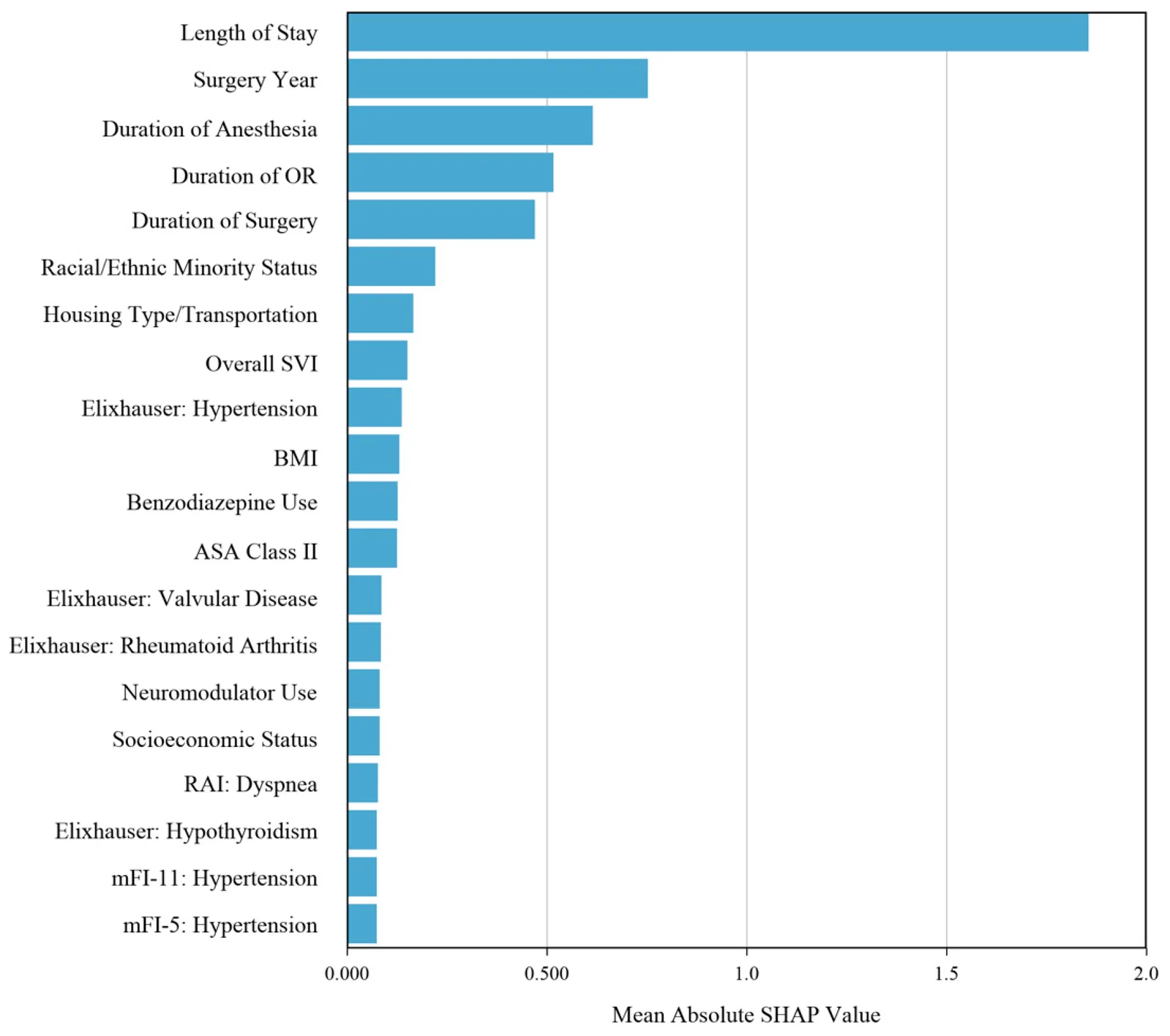
SHAP feature importance for healthcare-utilization prediction. Feature importance plot for the clinical-plus-SDH support vector regression model predicting the continuous healthcare-utilization score. The x-axis represents the mean absolute SHAP value, calculated as the average absolute contribution of each predictor to the model output. Larger values indicate greater average model attribution to model predictions.

SHAP analysis of the clinical-plus-SDH models demonstrated that SVI variables contributed to model output for readmission and HU (Figure 1 and Figure 2). Our analysis found that socioeconomic status was among the top contributors, along with length of stay, preoperative benzodiazepine use, age, and surgery year. For HU, racial and ethnic minority statuses, housing type and transportation, and overall SVI were among the top contributors along with length of stay, surgery year, and operative duration. Additional sensitivity analyses, including parsimonious clinical models, restricted-SVI models, deduplicated predictor sets, principal component analysis-based reduction, log-transformed HU modeling, recalibration assessment, domain-specific HU modeling, and temporal validation, did not alter the primary interpretation and are reported in the Supplementary Materials.

In domain-specific HU sensitivity analyses, imaging and procedures accounted for most of the total HU score (mean: 9.17 of 13.58 total HU points; Supplementary Figure S19). Across outpatient, acute care, referral, and imaging/procedure domains, adding SVI did not meaningfully improve MAE compared with matched clinical-only models, and no domain showed an incremental benefit from SVI. Temporal validation similarly supported the primary interpretation. In the primary temporal split, which trained through 2018 and tested in 2019 or later, the clinical-only logistic model for predicting readmission had an AUC of 0.618 compared with 0.574 for the clinical-plus-SDH model (Supplementary Figure S20). HU prediction also showed essentially no improvement with SVI addition. Results were consistent across all other prespecified temporal splits.

## 4. Discussion

SDHs have been well established as important factors influencing the prognosis of spine surgery. While prior studies in patients undergoing ACDF and lumbar fusion have noted important associations between the risk of postoperative complications and SDH domains such as race and socioeconomic status, PCDF cohorts have not been as extensively studied [10,11,12]. ML models have enabled higher-level data analysis and prediction among spine surgery cohorts, enabling the integration of several data categories and greater data volumes than traditional statistical methods. To our knowledge, this study is the first to employ various ML methods to evaluate whether SDH measures add predictive value beyond patient demographic, medical history, and additional clinical attributes for 90-day readmission and healthcare utilization (HU) specifically among PCDF patients.

In our single-institution cohort of 1015 patients undergoing PCDF, we found that both clinical-only and clinical-plus-SDH models outperformed noninformative baselines for readmission discrimination and HU prediction error, although overall performance was modest. The clinical-only logistic regression and random forest models performed best overall for readmission and HU respectively and adding SVI did not produce consistent improvement in model performance. However, SVI variables contributed to the joint clinical-plus-SDH models. This interpretation was unchanged in post hoc domain-specific HU analyses and temporal validation. Thus, SVI contributed to model attribution without demonstrating incremental predictive value.

The distinction between attribution and incremental prediction is significant when analyzing the prior literature. Prior studies have consistently shown that both clinical and social risk factors are associated with postoperative healthcare utilization across a series of varying methodologies. Gerlach et al. and Reyes et al. demonstrated the association between clinical and socioeconomic factors, such as minority and housing status, with postoperative healthcare utilization after spine surgery [4,11]. Rethorn et al. similarly noted that the presence of multiple SDH disparities at once, when analyzed with cluster modeling, offered more precise estimates of their impact on outcomes [13]. In parallel, the literature also supports the link between socioeconomic disadvantages and greater postoperative care needs. Opara et al. found that PCDF patients from “distressed” communities had significantly longer stays (6.24 vs. 3.92 days) and 2.3-fold higher odds of non-home discharge [14]. Similarly, a recent analysis of over 8500 spine surgeries concluded that SDH was associated with length of stay, discharge disposition, and 90-day readmission [5]. Altogether, our findings, similar to the literature, support SDH as a meaningful potential correlate for postoperative outcomes. However, these factors did not add incremental predictive value when incorporated into expansive clinical models.

Several factors likely contribute to this observed pattern. First, the relatively small number of readmission events (n = 157) compared to the number of candidate predictors (93 variables) limits stable estimation and reduces the model’s ability to detect incremental gains from the SDH predictor variables. Past studies looking at postoperative outcomes in complex spine cohorts have faced a similar issue, leading to poor generalizability and contributing to limited performance estimate [15,16]. Second, the clinical feature set already included strong preoperative and perioperative variables that likely captured the downstream information represented by SDH-related variables. Third, SVI themes may be correlated with one another and partly redundant with clinical variables. Altogether, these insights should not be interpreted as evidence that SVI is uninformative; rather, its predictive value is limited in the presence of detailed clinical data and small event count.

Our study further exemplifies both the advantage and constraints of ML methods in capturing complex interactions between SDH and clinical factors in spine surgery. Although ML models can capture complex interactions by making no assumptions about linearity, increasing algorithmic complexity did not improve performance within this cohort [4]. In our cohort, logistic regression performed best for readmission, while discrimination remained modest and HU models retained substantial prediction error. Prior cervical fusion research has similarly evaluated ML and regression approaches to predict readmission, finding large variation in the performance of ML models across clinical outcomes and restricted overall clinical utility [15,16]. To improve the interpretation of our models, the use of XAI with SHAP values was conducted. SHAP indicated the strength of select SVI themes and multiple clinical variables in most strongly affecting model output. Zaidat et al. similarly used SHAP to interpret an ML model for length of ICU stay after adult spinal deformity surgery, which indicated surgery duration and postoperative complications, such as infection, as the leading contributors [7]. It is important to note that SHAP values should not be interpreted as causal effects because a variable may receive substantial attribution within a joint model even when its inclusion does not improve performance. Nonetheless, such explainability is crucial if ML tools are to be trusted by clinicians. By improving risk stratification in PCDF populations, ML models may enable more targeted perioperative planning and resource allocation, ultimately supporting better patient-centered care.

Ultimately, our results reinforce that traditional clinical risk factors accounted for most of the predictive performance compared to social determinants in shaping outcomes. Prior spine studies suggest that selected postoperative utilization domains may be modified through targeted care pathways. Haffner et al. demonstrated that preoperative multimodal analgesia reduced early postoperative narcotic consumption. Pak et al. associated outpatient spine-clinic utilization with fewer emergency department visits, and Wu et al. described a readmission-reduction program integrating ML with workflow analysis and simulation [17,18,19]. These studies do not establish that SVI-directed interventions will improve outcomes, but they support prospective evaluation of modifiable care processes. Prospective studies should evaluate whether incorporating transportation support, discharge planning, and comorbidity management into perioperative pathways can reduce avoidable readmissions and resource use.

Despite several valuable conclusions, our study has certain limitations. This study is a retrospective analysis from a single academic health system, resulting in limited generalizability given a potential skew in patient population. Site-specific contributions and site-level adjustment could not be reported due to limitations in data collection and extraction. Complete-case analysis excluded 35 of 1050 patients possibly producing selection bias. All cohort readmissions recorded outside of our institution’s EMR were not included in data analysis, potentially resulting in undercounting of the primary outcome. Additionally, the cohort was recorded over a span of 20 years, resulting in possible changes in surgical technique, perioperative pathways, coding practices, insurance structures, discharge planning, and follow-up that may have influenced the results. Census-tract-level SVI scores were used as proxies for the individual patient, which may introduce ecological misclassification. Although SVI was linked using the address recorded at the time of surgery and temporally matched SVI releases, SVI is not measured annually, resulting in SVI values that may not capture neighborhood conditions at the precise time of surgery. Residential mobility during follow-up was also not captured.

Although domain-specific HU sensitivity analyses were performed, the primary total HU score remained an unweighted composite that assigns equal value to heterogeneous utilization events. Therefore, sparse domains such as acute care and referrals should be interpreted cautiously. Our analysis also does not capture the severity of complications or length of readmission beyond its occurrence. The number of readmission events was limited relative to the number of candidate predictors, increasing risk of model instability and overfitting despite repeated splits, regularization, and training-only hyperparameter tuning. Finally, repeated split and temporal sensitivity analyses do not replace external validation. Further validation in independent health systems is necessary to confirm these findings.

While we did include a comprehensive set of 93 variables, certain relevant factors—such as individual income, level of education, and detailed postoperative care adherence—were not captured. Certain clinically relevant variables were also not available with enough granularity, including detailed diagnosis distribution, myelopathy severity, revision status, and complication severity. It is also essential to note that despite the presence of certain SDH criteria as strong predictors of healthcare utilization, we cannot infer that they alone drive increased utilization, as multiple clinical and social factors likely interact to influence patient behavior. For example, it cannot be inferred that patients delayed seeking critical medical care solely due to isolated socioeconomic factors. Further analysis is required to determine the root cause of these patients’ limitations.

Our study opens several directions for further research. First, future studies should emphasize utilizing national database sources and prospective studies that incorporate thousands of real-time, patient-level SDH evaluations within PCDF perioperative care pathways. Future studies could also examine transportation planning, discharge coordination, social work screening, home health referral, and outpatient follow-up support, while determining whether these interventions reduce avoidable utilization without introducing inequitable treatment. Because utilization does not fully describe functional recovery, future studies should also pair HU with patient-level measures of disability, rehabilitation needs, and functional outcomes [16,20]. The further incorporation of ML and SDH into risk-stratification frameworks, such as ERAS and perioperative planning guidelines, has the potential to promote equity and efficiency in spine surgery care by decreasing the burden of avoidable readmission and resource use among high-risk populations.

## 5. Conclusions

The presented work illustrates that census-tract SVI measures contribute to ML model attribution for 90-day readmission and healthcare utilization after PCDF. However, adding SVI did not improve predictive performance beyond the clinical feature set. These findings suggest that SDH measures may provide contextual information alongside conventional clinical variables, but the evaluated models should be considered exploratory rather than clinically deployable. Future work should externally validate these findings, improve calibration, and determine whether SDH-informed interventions can reduce postoperative utilization and disparities after PCDF.

## Figures and Tables

**Table 1:** (**a**) Machine learning model variable list and (**b**) Healthcare-utilization score components.

(**a**)	
**Category**	**Variables ^1^**
Demographics and Baseline Characteristics	Age at procedure; body mass index; ASA class
Perioperative Factors	Index length of stay; surgery duration; operating-room duration; anesthesia duration; surgery year
Preoperative Medication Use	Opioid use within 60 days before surgery; benzodiazepine use within 60 days before surgery; preoperative neuromodulator use
Comorbidity Indices	Charlson Comorbidity Index, Elixhauser Comorbidity Index
Frailty Indices	mFI-5, mFI-11, RAI
Tobacco and Clinical Validation Variables	Former tobacco use, current tobacco use, myelopathy
Social Vulnerability Index: Key Categories	SVI theme 1: socioeconomic status percentile; SVI theme 2: household characteristics percentile; SVI theme 3: racial/ethnic minority status percentile; SVI theme 4: housing/transportation percentile; overall SVI percentile
(**b**)	
**Component**	**HU Component Variable**
1	Number of neurosurgery visits
2	Number of orthopedic visits
3	Number of primary care visits
4	Number of urgent care visits
5	Number of ICUs
6	Number of hospitalizations
7	Number of EDs
8	Number of PT referrals
9	Number of other referrals
10	Number of PT encounters
11	Number of imaging orders
12	Number of cardiac procedures
13	Number of venous duplex procedures
14	Number of drainage aspirations
15	Number of IVC filters
16	Number of invasive procedures
17	Number of pulmonary procedures
18	Number of EMG NCS procedures

^1^ Table 1 presents selected cohort characteristics and complete list can be found in Supplementary Table S7. Abbreviation list: ASA class: American Society of Anesthesiologists score; mFI-5: modified frailty index-5; mFI-11: modified frailty index-11; RAI: risk analysis index. HU = Healthcare utilization; ICU = intensive care unit; ED = emergency department; PT = physical therapy; IVC = inferior vena cava; EMG = electromyography; NCS = nerve conduction study. Component 9, “other referrals”, refers to specialty or ancillary referrals other than neurosurgery, orthopedic surgery, primary care, urgent care, or physical therapy referrals. Component 16, “invasive procedures”, refers to post-discharge invasive procedures not otherwise captured by cardiac procedures, venous duplex studies, drainage aspirations, IVC filter placement, pulmonary procedures, or EMG/NCS procedures.

**Table 2:** Model performance for 90-day readmission and healthcare utilization.

Panel A. 90-day readmission classification										
**Outcome**	**Feature set**	**Model**	**AUROC**	**AUPRC**		**Brier**		**Sensitivity**		**Specificity**
90-day readmission	Clinical Only	Logistic	0.646 [0.572, 0.736]	0.278 [0.205, 0.391]		0.226 [0.212, 0.245]		0.466 [0.330, 0.613]		0.752 [0.554, 0.869]
90-day readmission	Clinical Only	ENet	0.637 [0.552, 0.718]	0.272 [0.201, 0.394]		0.227 [0.212, 0.247]		0.446 [0.298, 0.613]		0.753 [0.604, 0.858]
90-day readmission	Clinical Only	BRF	0.615 [0.537, 0.703]	0.264 [0.198, 0.326]		0.225 [0.196, 0.242]		0.437 [0.233, 0.720]		0.726 [0.547, 0.848]
90-day readmission	Clinical Only	NN	0.608 [0.516, 0.696]	0.249 [0.179, 0.315]		0.194 [0.170, 0.220]		0.263 [0.129, 0.444]		0.863 [0.742, 0.929]
90-day readmission	Clinical Only	XGBoost	0.606 [0.519, 0.689]	0.250 [0.182, 0.337]		0.224 [0.198, 0.243]		0.991 [0.903, 1.000]		0.016 [0.000, 0.174]
90-day readmission	Clinical Only	Dummy	0.500 [0.500, 0.500]	0.153 [0.153, 0.153]		0.129 [0.129, 0.129]		0.000 [0.000, 0.000]		1.000 [1.000, 1.000]
90-day readmission	Clinical Only	Prevalence	0.500 [0.500, 0.500]	0.153 [0.153, 0.153]		0.129 [0.129, 0.129]		0.000 [0.000, 0.000]		1.000 [1.000, 1.000]
90-day readmission	Clinical Plus SDH	Logistic	0.635 [0.566, 0.725]	0.270 [0.199, 0.381]		0.227 [0.212, 0.247]		0.487 [0.298, 0.727]		0.709 [0.513, 0.839]
90-day readmission	Clinical Plus SDH	ENet	0.623 [0.547, 0.699]	0.265 [0.199, 0.374]		0.228 [0.213, 0.246]		0.444 [0.298, 0.638]		0.740 [0.532, 0.868]
90-day readmission	Clinical Plus SDH	BRF	0.597 [0.523, 0.670]	0.255 [0.185, 0.350]		0.229 [0.201, 0.242]		0.434 [0.233, 0.645]		0.714 [0.497, 0.836]
90-day readmission	Clinical Plus SDH	NN	0.595 [0.462, 0.671]	0.236 [0.148, 0.314]		0.197 [0.173, 0.243]		0.279 [0.129, 0.823]		0.828 [0.248, 0.917]
90-day readmission	Clinical Plus SDH	XGBoost	0.586 [0.496, 0.667]	0.236 [0.179, 0.349]		0.228 [0.194, 0.245]		0.998 [1.000, 1.000]		0.001 [0.000, 0.000]
90-day readmission	Clinical Plus SDH	Dummy	0.500 [0.500, 0.500]	0.153 [0.153, 0.153]		0.129 [0.129, 0.129]		0.000 [0.000, 0.000]		1.000 [1.000, 1.000]
90-day readmission	Clinical Plus SDH	Prevalence	0.500 [0.500, 0.500]	0.153 [0.153, 0.153]		0.129 [0.129, 0.129]		0.000 [0.000, 0.000]		1.000 [1.000, 1.000]
Panel B. Healthcare-utilization regression										
**Outcome**	**Feature set**	**Model**	**MAE**		**RMSE**		**R^2^**		**Median AE**	
Healthcare utilization	Clinical Only	RF	4.797 [4.187, 5.442]		7.369 [5.859, 8.731]		0.326 [0.060, 0.482]		3.369 [2.895, 3.921]	
Healthcare utilization	Clinical Only	XGBoost	4.805 [4.196, 5.449]		7.425 [5.871, 8.955]		0.320 [0.172, 0.475]		3.352 [2.901, 3.791]	
Healthcare utilization	Clinical Only	SVR	4.815 [4.236, 5.362]		7.766 [5.987, 8.996]		0.258 [0.147, 0.376]		3.179 [2.860, 3.604]	
Healthcare utilization	Clinical Only	Dummy	5.279 [4.819, 5.779]		9.180 [7.529, 11.096]		−0.031 [−0.043, −0.021]		3.040 [3.000, 3.775]	
Healthcare utilization	Clinical Plus SDH	SVR	4.838 [4.261, 5.375]		7.846 [6.092, 9.178]		0.244 [0.134, 0.340]		3.163 [2.883, 3.562]	
Healthcare utilization	Clinical Plus SDH	RF	4.840 [4.213, 5.453]		7.425 [5.860, 8.776]		0.317 [0.127, 0.479]		3.426 [3.034, 3.947]	
Healthcare utilization	Clinical Plus SDH	XGBoost	4.867 [4.325, 5.527]		7.575 [5.991, 9.116]		0.291 [0.068, 0.456]		3.389 [2.946, 3.876]	
Healthcare utilization	Clinical Plus SDH	Dummy	5.279 [4.819, 5.779]		9.180 [7.529, 11.096]		−0.031 [−0.043, −0.021]		3.040 [3.000, 3.775]	

Values are mean (2.5th, 97.5th percentile) across repeated test splits. Higher AUROC, AUPRC, sensitivity, specificity, and R^2^ indicate better performance; lower Brier score, MAE, RMSE, and median absolute error indicate better performance.

**Table 3:** Selected descriptive comparisons by 90-day readmission and healthcare-utilization status.

Panel A. Selected clinical and perioperative characteristics by 90-day readmission							
Characteristic	Overall	No readmission	Readmission	*p*-value	Effect size	FDR q	Effect size 95% CI
Age, years	64.7 (11.2)	64.5 (11.2)	65.9 (11.1)	0.159	0.12	0.381	[−0.05, 0.29]
Body mass index, kg/m^2^	29.1 (7.3)	29.0 (7.4)	29.7 (6.9)	0.244	0.1	0.487	[−0.07, 0.27]
Surgery duration, hours	4.4 (2.4)	4.3 (2.4)	4.6 (2.1)	0.175	0.11	0.395	[−0.06, 0.28]
Operating-room duration, hours	5.8 (2.1)	5.7 (2.1)	6.2 (2.1)	0.012	0.22	0.055	[0.05, 0.39]
Anesthesia duration, hours	5.9 (1.9)	5.8 (1.9)	6.3 (2.2)	0.006	0.27	0.035	[0.10, 0.44]
Index length of stay, days	7.2 (6.3)	6.7 (5.7)	10.0 (8.6)	<0.001	0.52	<0.001	[0.35, 0.69]
ASA class >=3	752 (74.1%)	625 (72.8%)	127 (80.9%)	0.012	0.09	0.055	[0.01, 0.12]
Multilevel PCDF	987 (97.2%)	833 (97.1%)	154 (98.1%)	0.605	0.07	0.758	[−0.11, 0.23]
Instrumentation	996 (98.1%)	847 (98.7%)	149 (94.9%)	0.005	−0.22	0.033	[−0.45, −0.11]
Myelopathy	43 (4.2%)	40 (4.7%)	3 (1.9%)	0.116	−0.15	0.322	[−0.31, 0.03]
Current tobacco use	19 (1.9%)	17 (2.0%)	2 (1.3%)	0.754	−0.06	0.875	[−0.22, 0.12]
Congestive heart failure	57 (5.6%)	39 (4.5%)	18 (11.5%)	<0.001	0.25	0.006	[0.13, 0.47]
Cardiac arrhythmia	100 (9.9%)	79 (9.2%)	21 (13.4%)	0.107	0.13	0.322	[−0.03, 0.31]
Chronic pulmonary disease	158 (15.6%)	127 (14.8%)	31 (19.7%)	0.116	0.13	0.322	[−0.03, 0.31]
Diabetes without complications	203 (20.0%)	167 (19.5%)	36 (22.9%)	0.318	0.08	0.545	[−0.08, 0.26]
Renal failure	83 (8.2%)	61 (7.1%)	22 (14.0%)	0.004	0.22	0.033	[0.08, 0.42]
Obesity	79 (7.8%)	64 (7.5%)	15 (9.6%)	0.368	0.08	0.575	[−0.09, 0.25]
Depression	193 (19.0%)	171 (19.9%)	22 (14.0%)	0.082	−0.16	0.296	[−0.32, 0.02]
SVI socioeconomic status theme percentile	0.4 (0.3)	0.4 (0.3)	0.4 (0.3)	0.526	0.05	0.728	[−0.12, 0.22]
SVI household characteristics theme percentile	0.3 (0.3)	0.3 (0.3)	0.3 (0.3)	0.989	0.0	1.000	[−0.17, 0.17]
SVI racial/ethnic minority status theme percentile	0.5 (0.2)	0.5 (0.2)	0.5 (0.2)	0.567	0.05	0.755	[−0.12, 0.22]
SVI housing/transportation theme percentile	0.4 (0.3)	0.4 (0.3)	0.4 (0.3)	0.895	0.01	1.000	[−0.16, 0.18]
Overall Social Vulnerability Index percentile	0.4 (0.4)	0.4 (0.4)	0.4 (0.3)	0.971	0.0	1.000	[−0.17, 0.17]
Panel B. Selected clinical and perioperative characteristics by healthcare-utilization group							
Characteristic	Overall	At/below-median HU	Above-median HU	*p*-value	Effect size	FDR q	Effect size 95% CI
Age, years	64.7 (11.2)	63.8 (11.0)	66.0 (11.2)	0.002	0.2	0.013	[0.07, 0.32]
Body mass index, kg/m^2^	29.1 (7.3)	29.2 (8.0)	29.0 (6.3)	0.540	−0.04	0.809	[−0.16, 0.09]
Surgery duration, hours	4.4 (2.4)	4.2 (2.5)	4.5 (2.2)	0.023	0.14	0.083	[0.02, 0.27]
Operating-room duration, hours	5.8 (2.1)	5.5 (2.0)	6.1 (2.1)	<0.001	0.28	<0.001	[0.16, 0.41]
Anesthesia duration, hours	5.9 (1.9)	5.6 (1.7)	6.3 (2.1)	<0.001	0.34	<0.001	[0.21, 0.46]
Index length of stay, days	7.2 (6.3)	6.0 (4.4)	9.0 (8.0)	<0.001	0.48	<0.001	[0.35, 0.61]
ASA class >=3	752 (74.1%)	420 (71.3%)	332 (77.9%)	0.016	0.09	0.063	[0.01, 0.14]
Multilevel PCDF	987 (97.2%)	571 (96.9%)	416 (97.7%)	0.496	0.04	0.777	[−0.08, 0.17]
Instrumentation	996 (98.1%)	582 (98.8%)	414 (97.2%)	0.059	−0.12	0.193	[−0.25, 0.00]
Myelopathy	43 (4.2%)	30 (5.1%)	13 (3.1%)	0.111	−0.1	0.307	[−0.23, 0.02]
Current tobacco use	19 (1.9%)	13 (2.2%)	6 (1.4%)	0.354	−0.06	0.704	[−0.18, 0.07]
Congestive heart failure	57 (5.6%)	22 (3.7%)	35 (8.2%)	0.002	0.19	0.013	[0.07, 0.32]
Cardiac arrhythmia	100 (9.9%)	44 (7.5%)	56 (13.1%)	0.003	0.19	0.014	[0.07, 0.32]
Chronic pulmonary disease	158 (15.6%)	75 (12.7%)	83 (19.5%)	0.003	0.18	0.015	[0.06, 0.31]
Diabetes without complications	203 (20.0%)	119 (20.2%)	84 (19.7%)	0.849	−0.01	0.962	[−0.14, 0.11]
Renal failure	83 (8.2%)	41 (7.0%)	42 (9.9%)	0.096	0.1	0.289	[−0.02, 0.23]
Obesity	79 (7.8%)	46 (7.8%)	33 (7.7%)	0.970	−0.0	0.998	[−0.13, 0.12]
Depression	193 (19.0%)	114 (19.4%)	79 (18.5%)	0.745	−0.02	0.962	[−0.15, 0.10]
SVI socioeconomic status theme percentile	0.4 (0.3)	0.4 (0.3)	0.4 (0.3)	0.855	0.01	0.962	[−0.11, 0.14]
SVI household characteristics theme percentile	0.3 (0.3)	0.3 (0.3)	0.3 (0.3)	0.824	0.01	0.962	[−0.11, 0.14]
SVI racial/ethnic minority status theme percentile	0.5 (0.2)	0.5 (0.2)	0.5 (0.2)	0.339	0.06	0.704	[−0.06, 0.19]
SVI housing/transportation theme percentile	0.4 (0.3)	0.4 (0.3)	0.4 (0.3)	0.378	0.06	0.704	[−0.07, 0.18]
Overall Social Vulnerability Index percentile	0.4 (0.4)	0.4 (0.4)	0.4 (0.3)	0.918	0.01	0.972	[−0.12, 0.13]

Continuous variables are mean (SD); categorical variables are n (%). Effect sizes are Cohen d for continuous variables, Cramer’s V for ASA class, and standardized mean difference for binary variables. *p*-values are unadjusted; FDR q values account for multiple comparisons within the descriptive table.

**Table 4:** Demographics for PCDF cohort.

**Age (Years, SD)**	64.7 (11.2)
**Body Mass Index (kg/m^2^), SD**	29.1 (7.3)
**Sex, n (%)**	
Male	599 (59.0)
Female	416 (41.0)
**PCDF Level, n (%)**	
Single	28 (2.8)
Multilevel	987 (97.2)
**ASA Class, n (%)**	
1	6 (0.6)
2	257 (25.3)
≥3	752 (74.1)
**30-day Readmission n (%)**	111 (10.9)
**90-day Readmission, n (%)**	157 (15.5)
**Healthcare Utilization (SD)**	13.6 (9.0)

## Data Availability

The deidentified raw data supporting the conclusions of this article will be made available by the authors on request.

## Supplementary Materials

The following supporting information can be downloaded at https://www.mdpi.com/article/10.3390/jcm15187047/s1, Figure S1, ROC and precision-recall curves for 90-day readmission; Figure S2, AUROC forest plot comparing clinical-only and clinical-plus-SDH readmission models; Figure S3, MAE forest plot comparing clinical-only and clinical-plus-SDH healthcare-utilization models; Figure S4, Predicted versus observed healthcare-utilization score; Figure S5, Paired incremental performance deltas for readmission after adding SVI; Figure S6, Paired incremental performance deltas for healthcare utilization after adding SVI; Figure S7, Calibration plot for 90-day readmission prediction; Figure S8, Decision-curve analysis for 90-day readmission prediction; Figure S9, Raw versus nested recalibrated calibration for readmission prediction; Figure S10, SHAP beeswarm plot for the clinical-plus-SDH readmission model; Figure S11, SHAP beeswarm plot for the clinical-plus-SDH healthcare-utilization model; Figure S12, SVI dependence panel for readmission prediction; Figure S13, SVI dependence panel for healthcare-utilization prediction; Figure S14, Parsimonious clinical baseline model performance for readmission; Figure S15, Parsimonious clinical baseline model performance for healthcare utilization; Figure S16, Sensitivity overview of paired incremental deltas; Figure S17, Deduplicated-predictor sensitivity analysis for readmission; Figure S18, Log-transformed healthcare-utilization sensitivity analysis; Figure S19, Incremental predictive value of SVI across healthcare-utilization domains; Figure S20, Forward-in-time temporal validation of incremental SVI predictive value; Table S1, Full 90-day readmission model performance; Table S2, Full healthcare-utilization model performance; Table S3, Parsimonious clinical baseline performance for 90-day readmission; Table S4, Parsimonious clinical baseline performance for healthcare utilization; Table S5, Reconstructed cohort flow for complete-case analysis; Table S6, Variables with missingness among eligible encounters before complete-case restriction; Table S7, Full predictor variable list for final leakage-free models; Table S8, Paired incremental comparison of clinical-plus-SDH versus clinical-only models; Table S9, Post-hoc recalibration sensitivity for the clinical-plus-SDH logistic model; Table S10, Sensitivity analyses for restricted SVI and PCA specifications; Table S11, SVI theme ranks from SHAP attribution; Table S12, Hyperparameter search spaces and tuning protocol; Table S13, TRIPOD + AI checklist for the revised PCDF prediction-model manuscript; Table S14, Healthcare-utilization domain definitions used for domain-specific sensitivity analyses.
