# Supplementary material for: Predictive Analysis of Social Determinants of Health in Posterior Cervical Spine Surgery
Source: J Clin Med. 2026 Sep 11;15(18):7047. doi: 10.3390/jcm15187047 (PMC13607260; doi:10.3390/jcm15187047)
Supplement: Supplementary file 1 [file jcm-15-07047-s001.zip › jcm-4416635-supplementary.pdf]

Supplementary Figures

Predictive Analysis of Social Determinants of Health in Posterior Cervical Spine Surgery

General note: AUROC, area under the receiver operating characteristic curve; AUPRC, area under the precision-recall curve; ECE, expected calibration error; HU, healthcare utilization; MAE, mean absolute error; PCA, principal component analysis; SDH, social determinants of health; SHAP, Shapley Additive Explanations; SVI, Social Vulnerability Index.

Supplementary Figure S1. ROC and precision-recall curves for 90-day readmission.

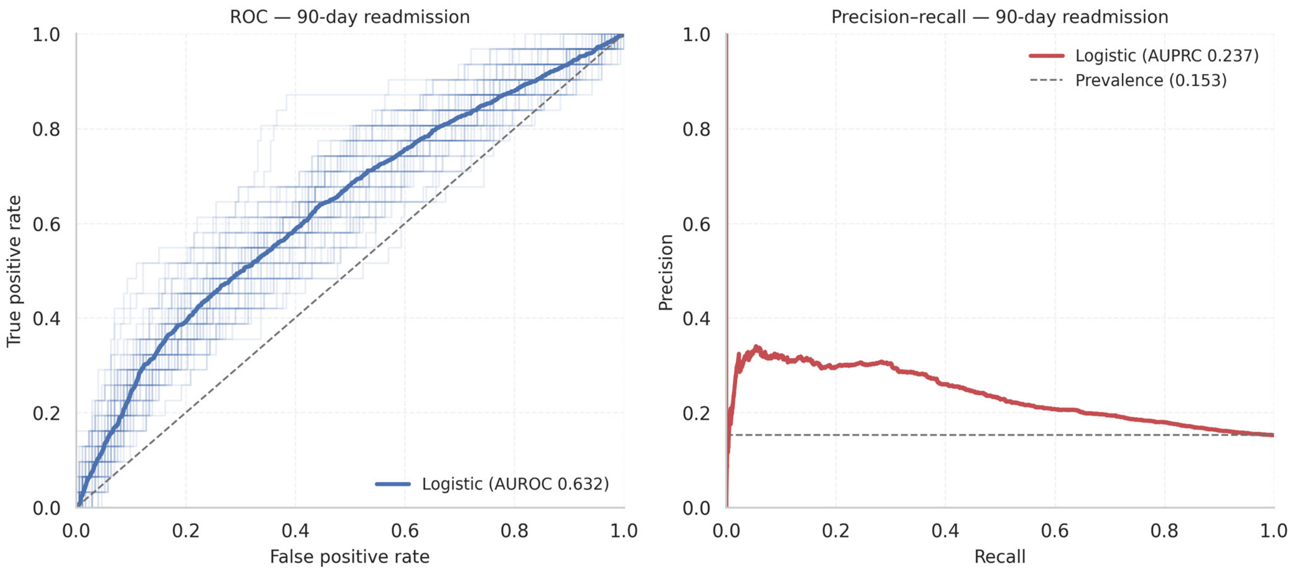

Note. ROC and precision-recall curves summarize discrimination for 90-day post-discharge readmission models. AUPRC should be interpreted relative to the observed readmission prevalence.

Supplementary Figure S2. AUROC forest plot comparing clinical-only and clinical-plus-SDH readmission models.

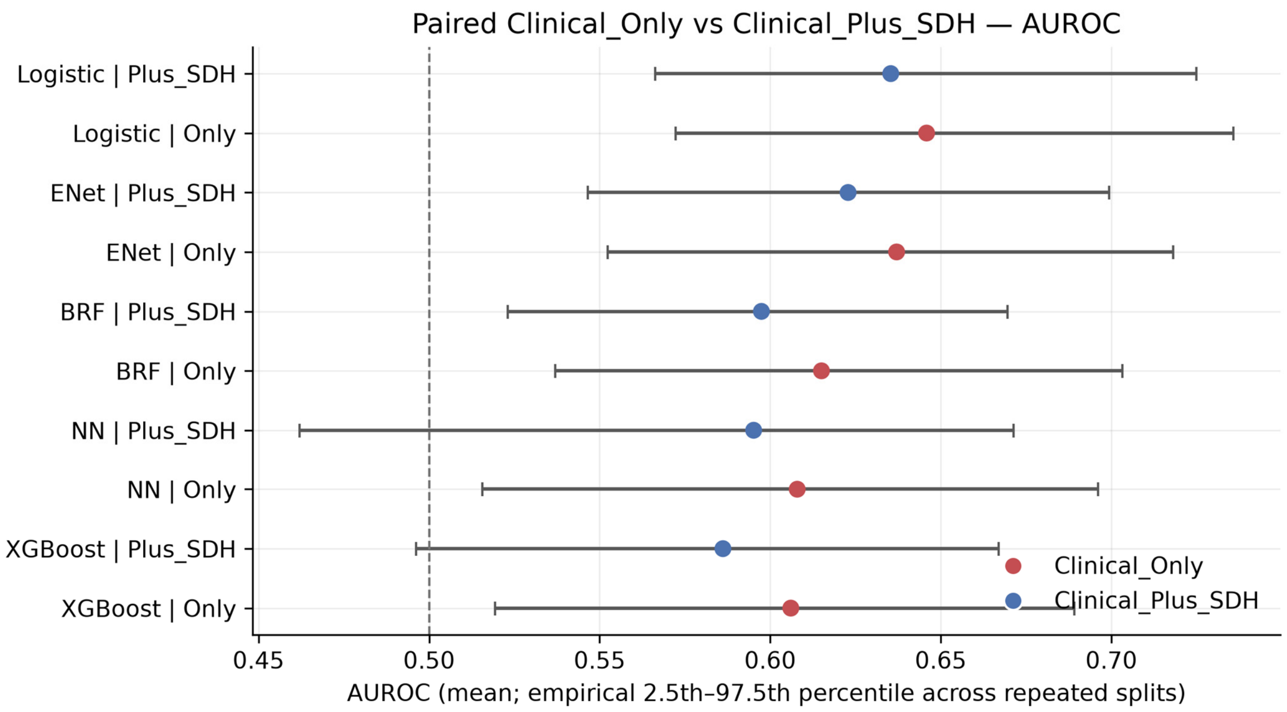

Note. Points represent mean AUROC across repeated test splits; intervals show empirical 2.5th to 97.5th percentiles.

Supplementary Figure S3. MAE forest plot comparing clinical-only and clinical-plus-SDH healthcare-utilization models.

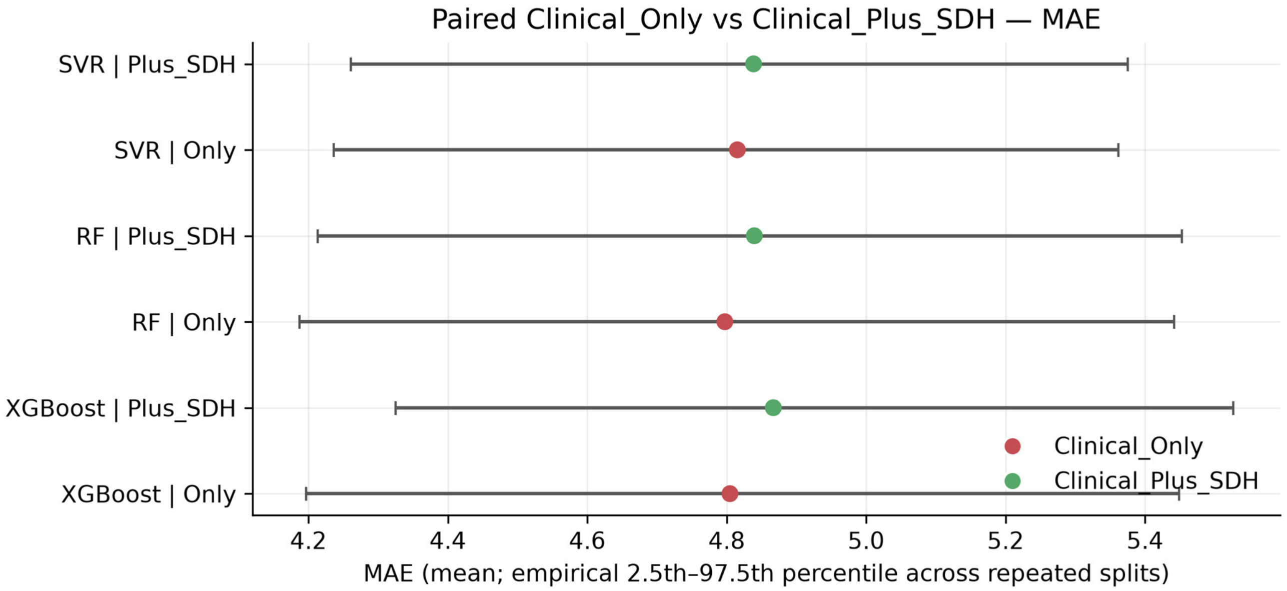

Note. Points represent mean MAE across repeated test splits; lower values indicate better prediction. Intervals show empirical 2.5th to 97.5th percentiles.

Supplementary Figure S4. Predicted versus observed healthcare-utilization score.

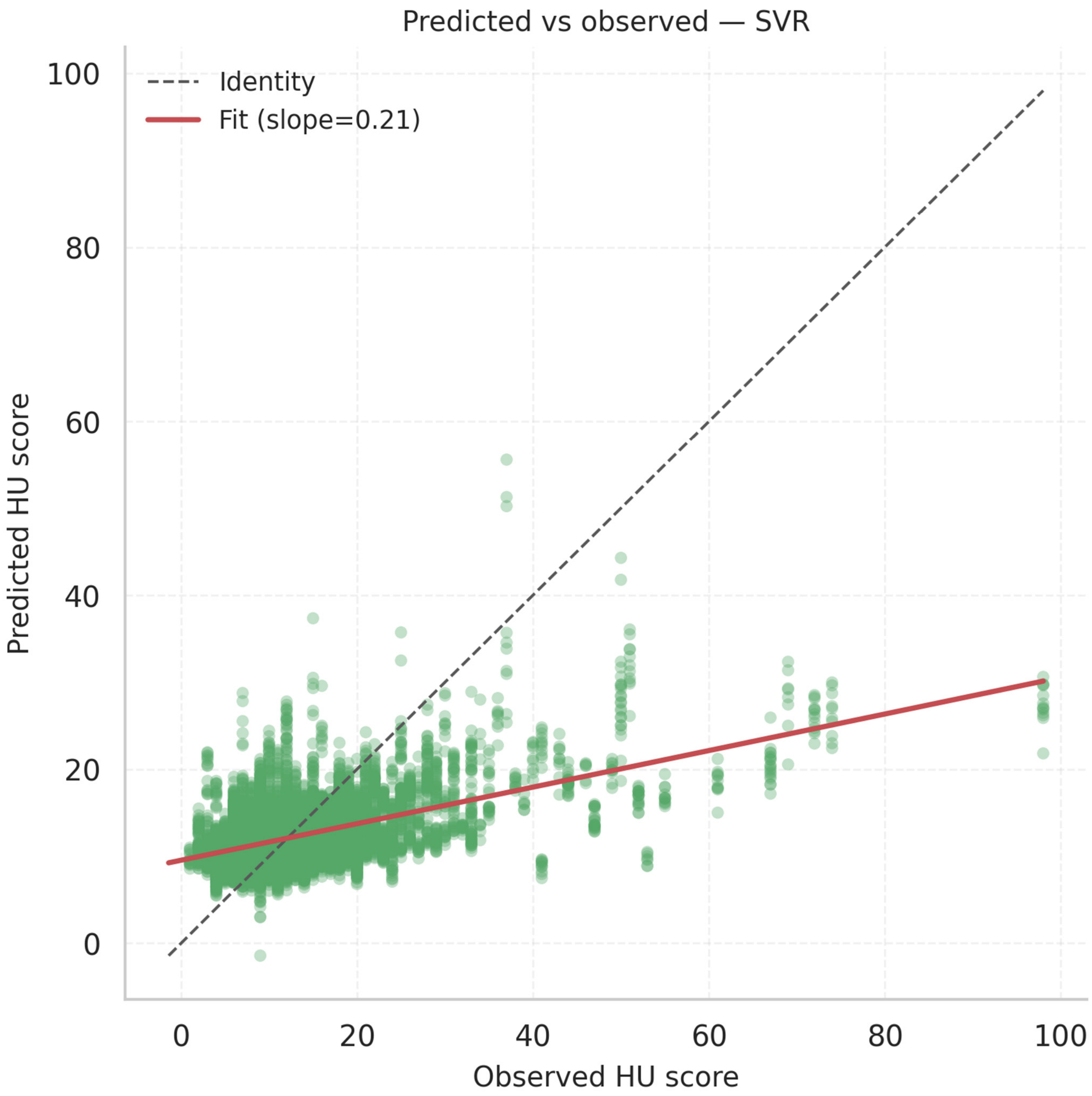

Note. The dashed line represents perfect agreement between observed and predicted HU score.

Supplementary Figure S5. Paired incremental performance deltas for readmission after adding SVI.

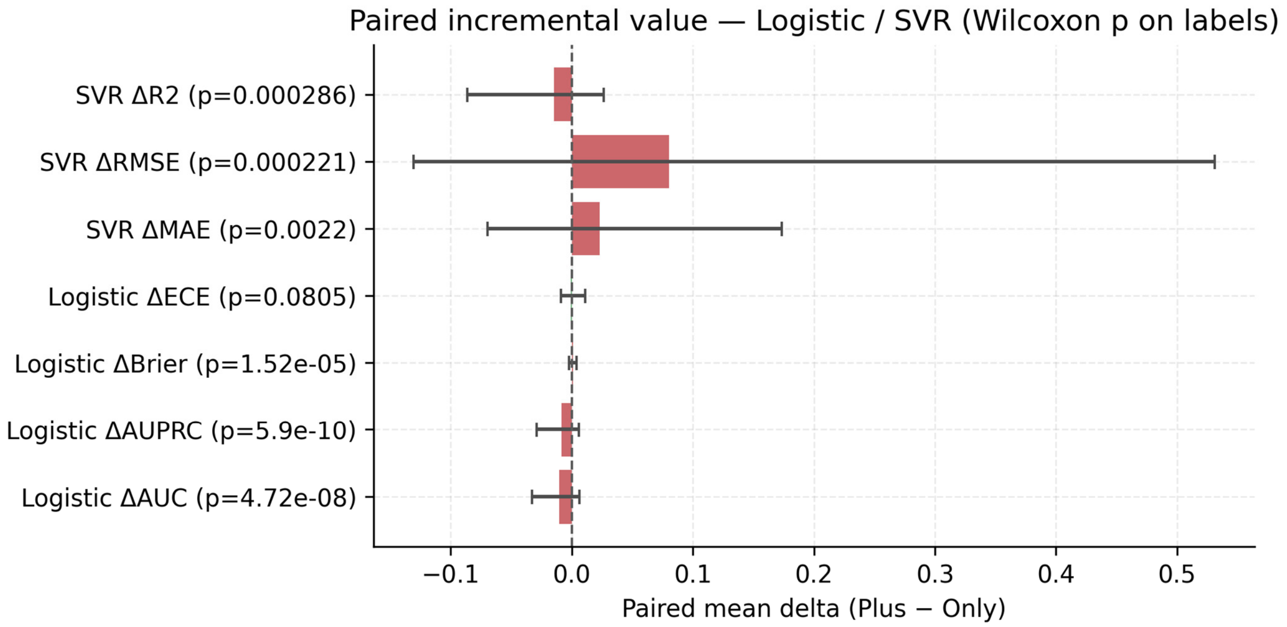

Note. Paired deltas compare clinical-plus-SDH models with corresponding clinical-only models within repeated splits. Positive deltas do not uniformly indicate improvement across all metrics.

Supplementary Figure S6. Paired incremental performance deltas for healthcare utilization after adding SVI.

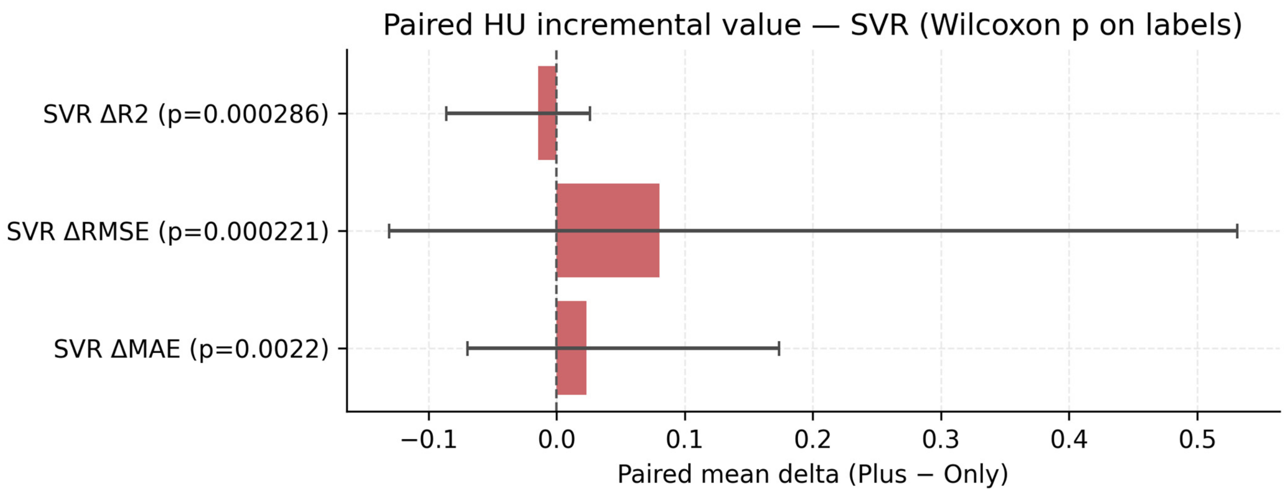

Note. Paired deltas compare clinical-plus-SDH models with corresponding clinical-only models within repeated splits. For MAE and RMSE, lower values indicate better performance.

Supplementary Figure S7. Calibration plot for 90-day readmission prediction.

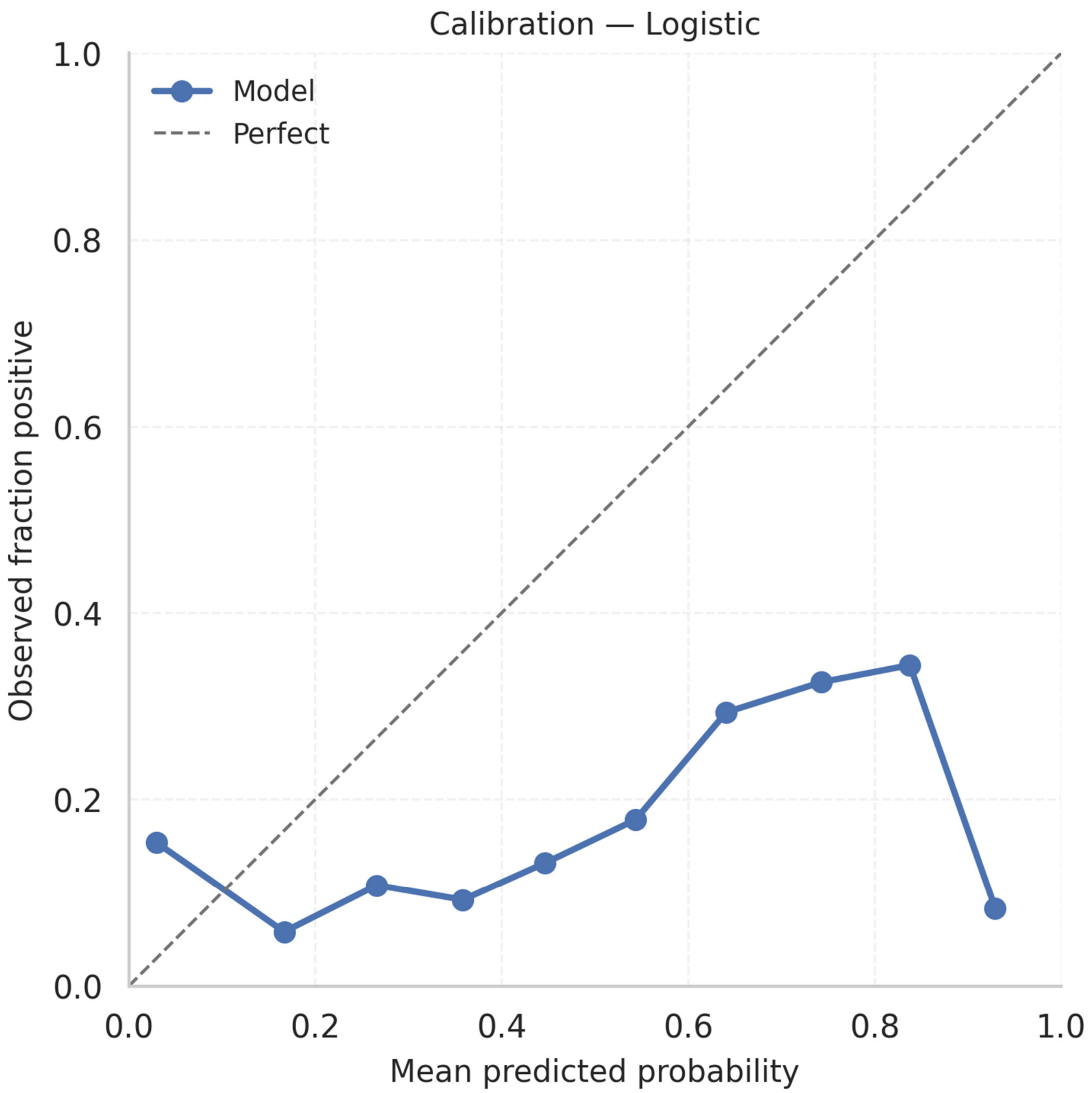

Note. Calibration displays agreement between predicted and observed readmission risk for the classification models.

Supplementary Figure S8. Decision-curve analysis for 90-day readmission prediction.

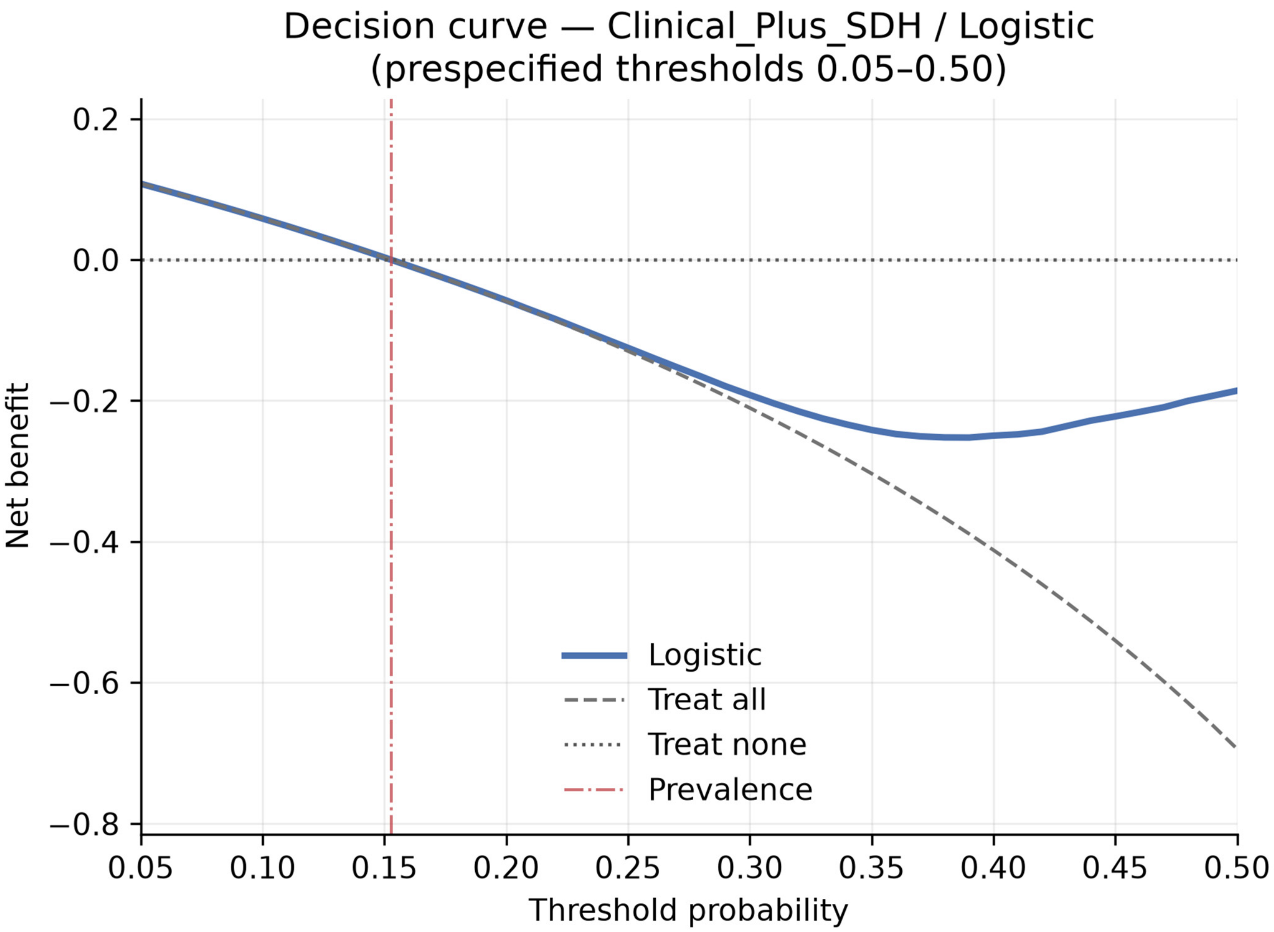

Note. Net benefit is shown across clinically plausible readmission-risk thresholds. Results should be interpreted as exploratory and not as evidence of clinical deployability.

Supplementary Figure S9. Raw versus nested recalibrated calibration for readmission prediction.

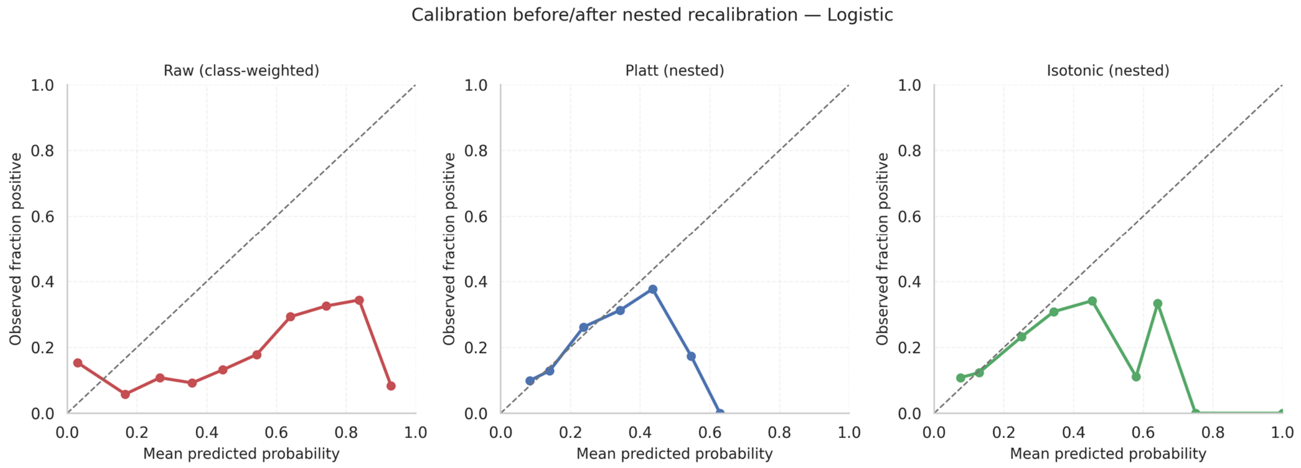

Note. Platt and isotonic recalibration were evaluated as post-hoc sensitivity analyses to assess probability calibration after class-weighted modeling.

Supplementary Figure S10. SHAP beeswarm plot for the clinical-plus-SDH readmission model.

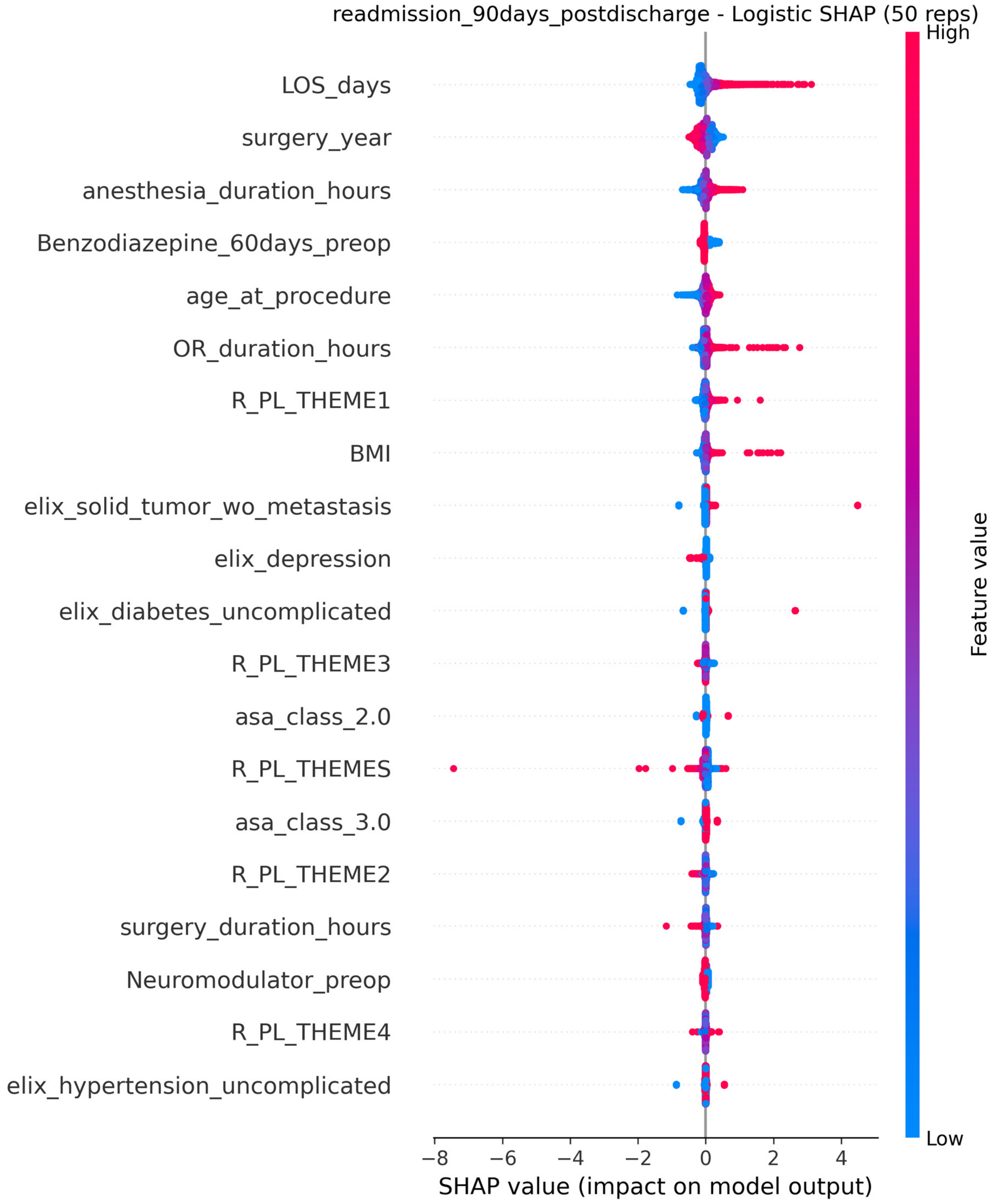

Note. SHAP values describe model-specific attribution and should not be interpreted as causal effects.

Supplementary Figure S11. SHAP beeswarm plot for the clinical-plus-SDH healthcare-utilization model.

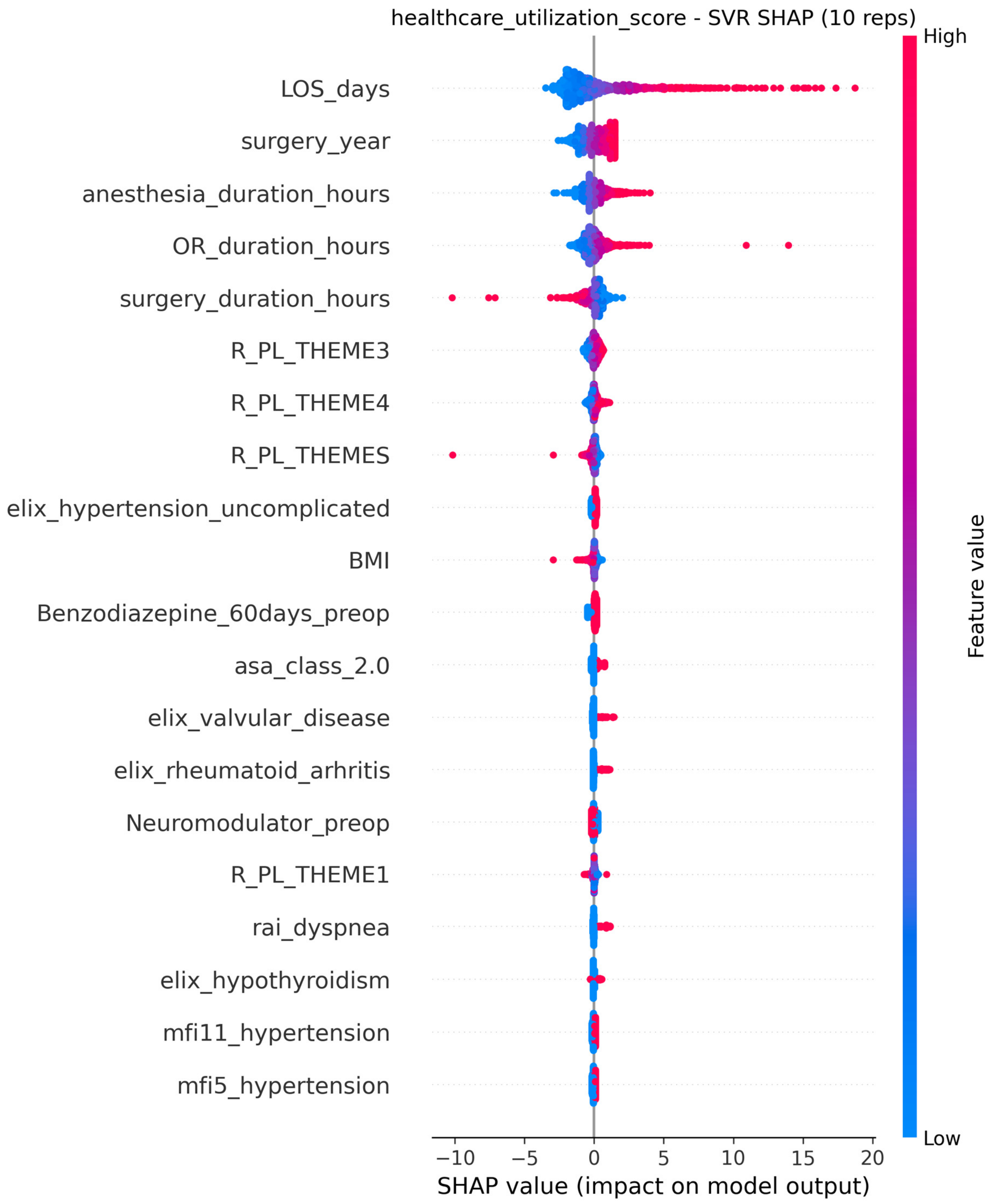

Note. SHAP values describe model-specific attribution and should not be interpreted as causal effects.

Supplementary Figure S12. SVI dependence panel for readmission prediction.

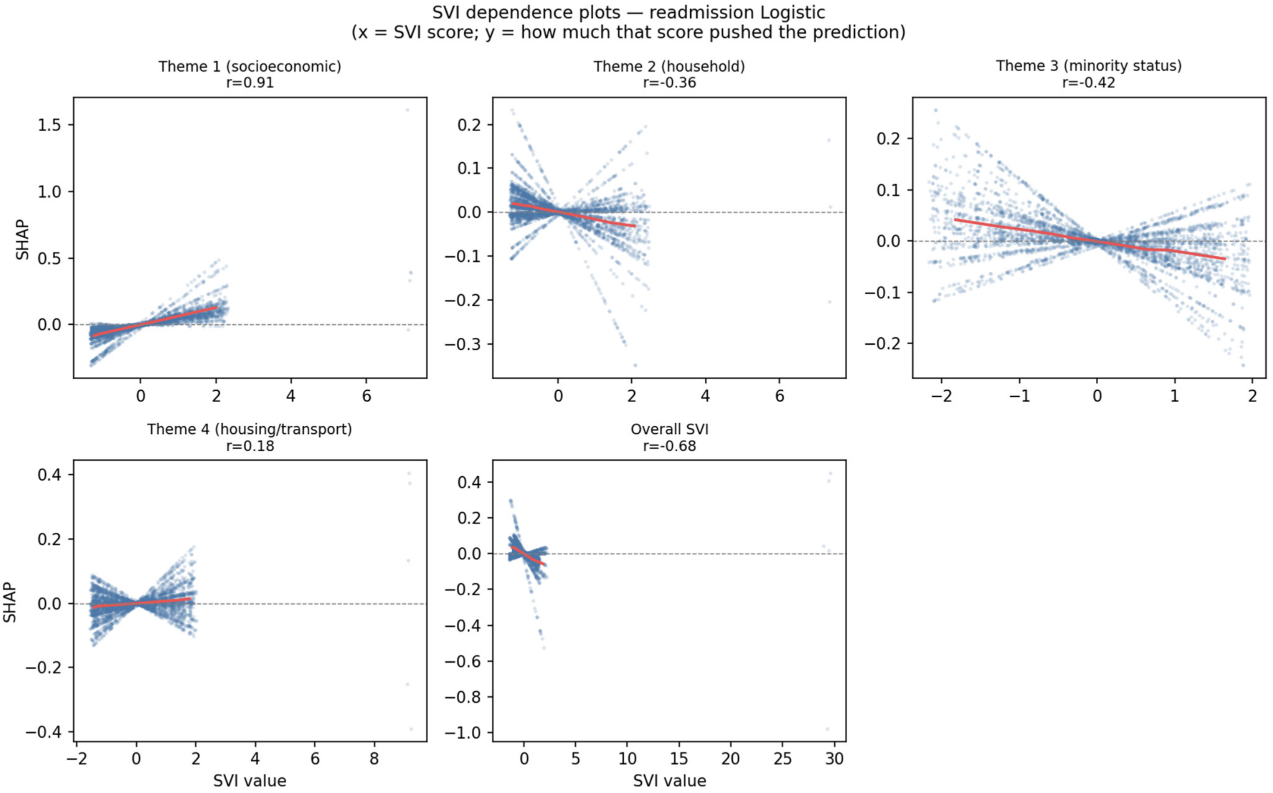

Note. Dependence plots evaluate how SVI values relate to SHAP attribution within the fitted readmission model.

Supplementary Figure S13. SVI dependence panel for healthcare-utilization prediction.

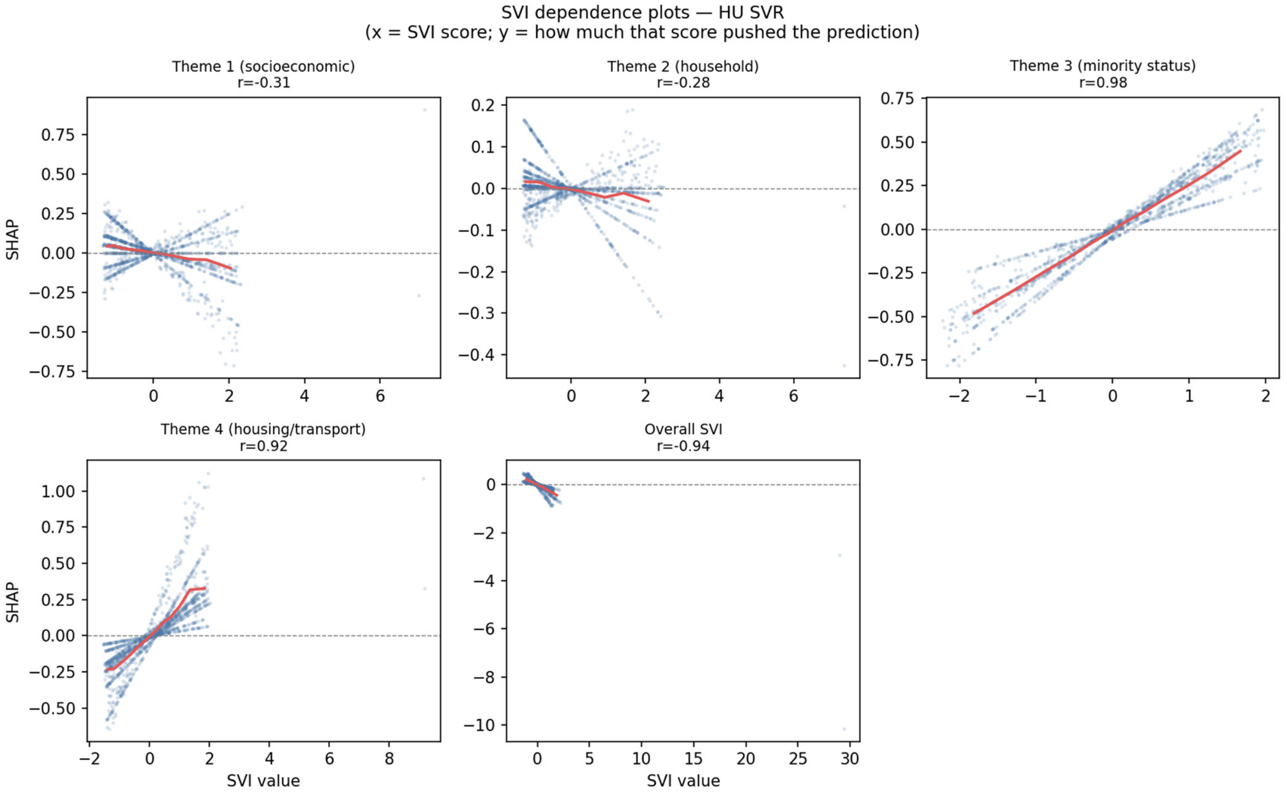

Note. Dependence plots evaluate how SVI values relate to SHAP attribution within the fitted HU model.

Supplementary Figure S14. Parsimonious clinical baseline model performance for readmission.

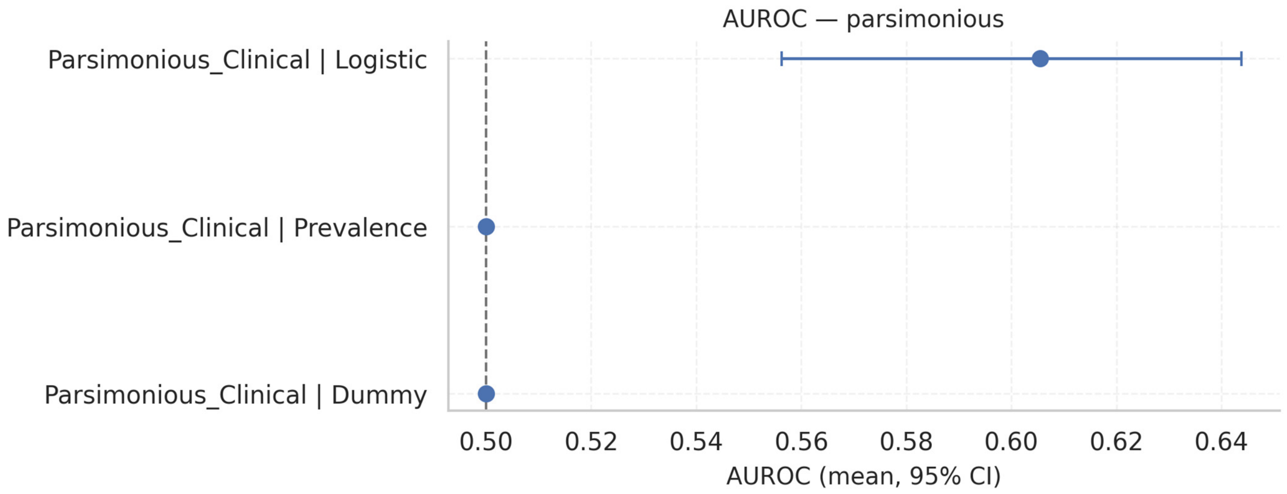

Note. The parsimonious model used standard clinical predictors to benchmark whether more complex models added meaningful value.

Supplementary Figure S15. Parsimonious clinical baseline model performance for healthcare utilization.

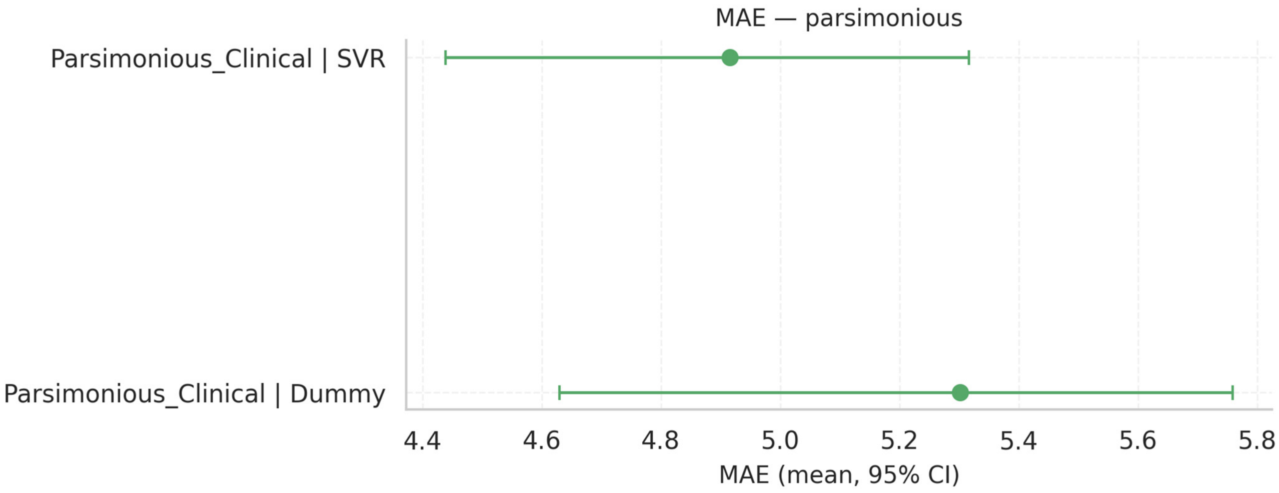

Note. The parsimonious model used standard clinical predictors to benchmark whether more complex models added meaningful value.

Supplementary Figure S16. Sensitivity overview of paired incremental deltas.

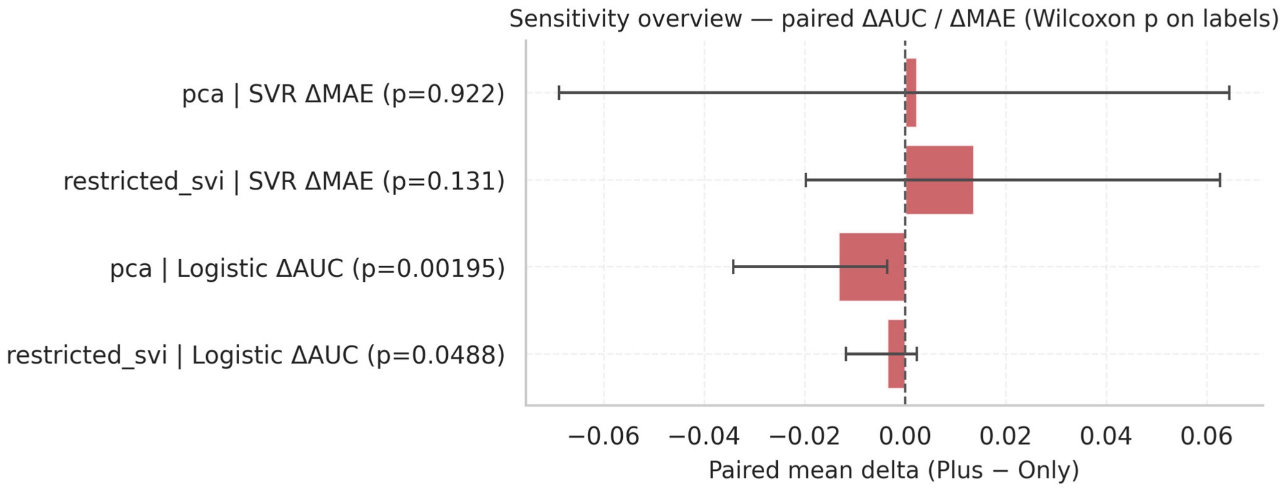

Note. Sensitivity analyses assess whether the primary interpretation changes under alternative feature or modeling strategies.

Supplementary Figure S17. Deduplicated-predictor sensitivity analysis for readmission.

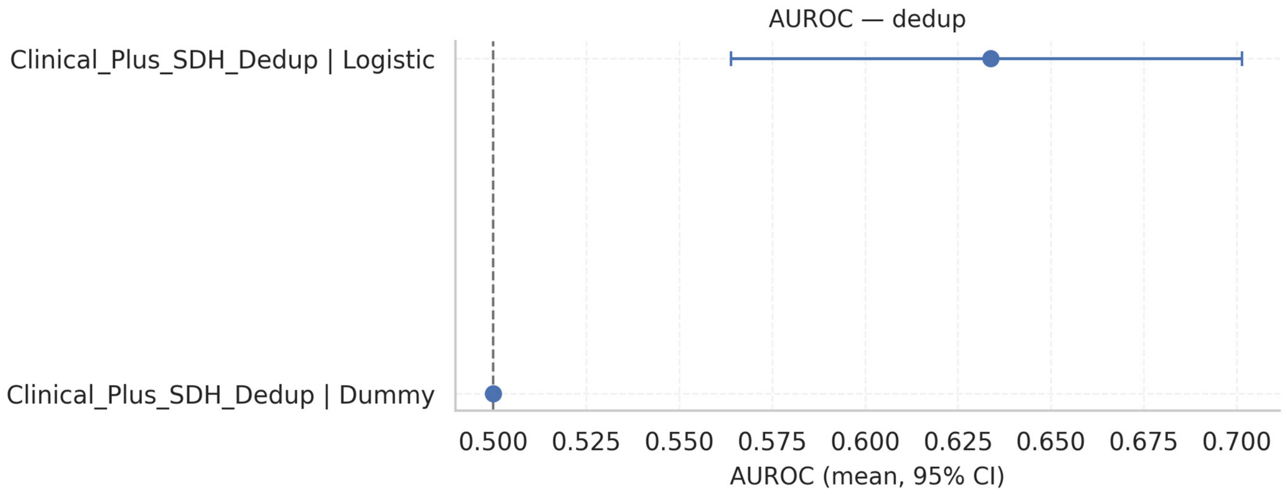

Note. This sensitivity analysis addresses collinearity and duplicate clinical-condition indicators across comorbidity/frailty measures.

Supplementary Figure S18. Log-transformed healthcare-utilization sensitivity analysis.

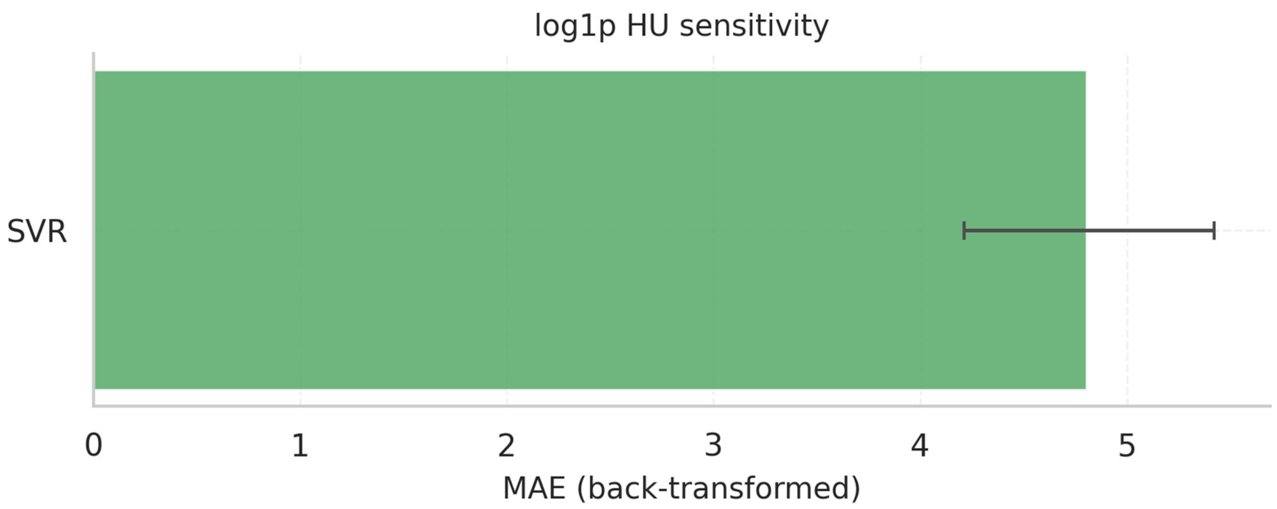

Note. This sensitivity analysis evaluates whether modeling log-transformed HU changes prediction error on the original HU scale.

Supplementary Figure S19. Incremental Predictive Value of SVI Across Healthcare-Utilization Domains

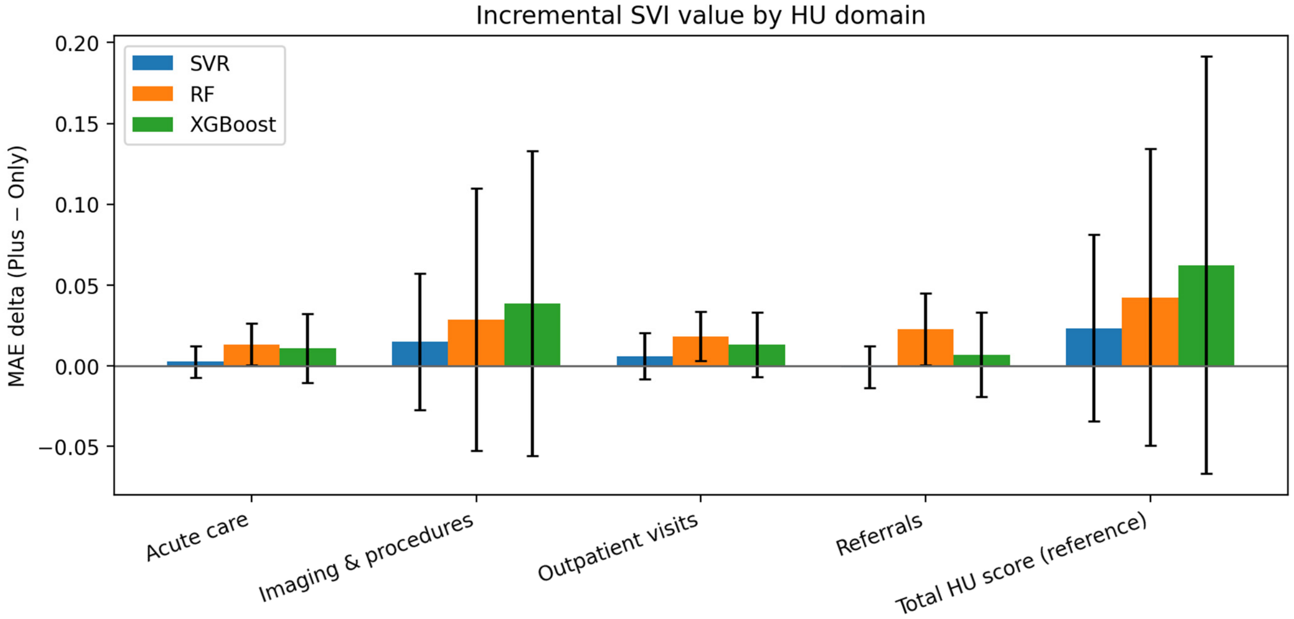

Supplementary Figure S20. Forward-in-Time Temporal Validation of Incremental SVI Predictive Value

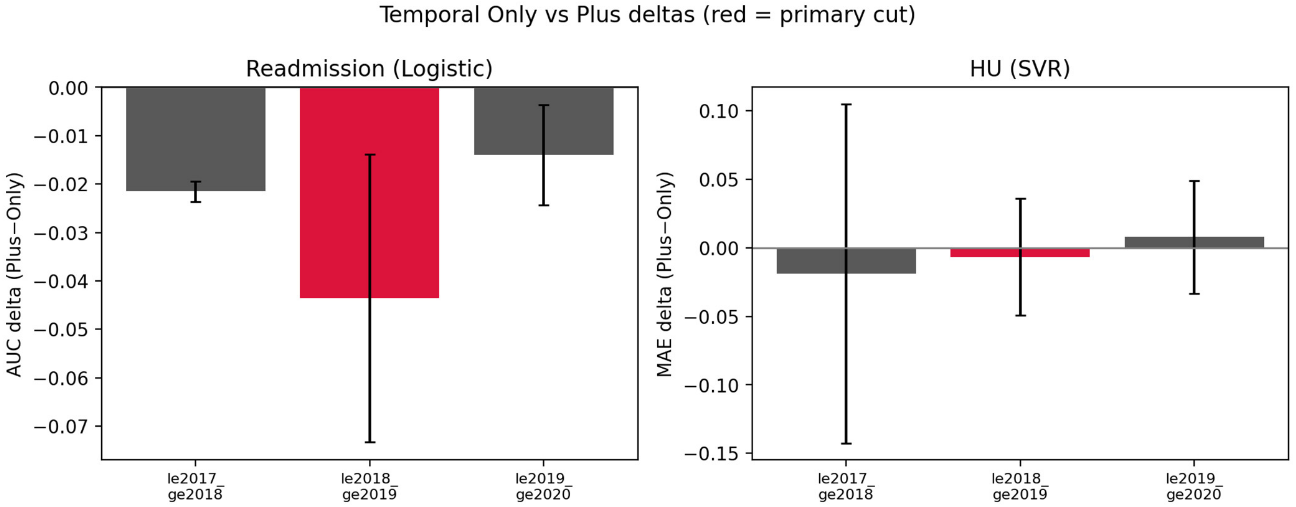

Supplementary Tables

PCDF social vulnerability and machine-learning revision

Note. Unless otherwise specified, bracketed intervals are empirical 2.5th to 97.5th percentile ranges across repeated train-test splits. AUROC = area under the receiver operating characteristic curve; AUPRC = area under the precision-recall curve; ECE = expected calibration error; HU = healthcare utilization; MAE = mean absolute error; RMSE = root mean squared error; SDH = social determinants of health; SVI = Social Vulnerability Index.

Supplementary Table S1. Full 90-day readmission model performance.

Feature set	Model	AUROC	AUPRC	Brier	Accuracy	Sensitivity	Specificity	PPV	NPV
Clinical_Only	BRF	0.615 [0.537, 0.703]	0.264 [0.198, 0.326]	0.225 [0.196, 0.242]	0.682 [0.569, 0.770]	0.437 [0.233, 0.720]	0.726 [0.547, 0.848]	0.226 [0.178, 0.293]	0.879 [0.856, 0.915]
Clinical_Only	Dummy	0.500 [0.500, 0.500]	0.153 [0.153, 0.153]	0.129 [0.129, 0.129]	0.847 [0.847, 0.847]	0.000 [0.000, 0.000]	1.000 [1.000, 1.000]		0.847 [0.847, 0.847]
Clinical_Only	ENet	0.637 [0.552, 0.718]	0.272 [0.201, 0.394]	0.227 [0.212, 0.247]	0.707 [0.597, 0.787]	0.446 [0.298, 0.613]	0.753 [0.604, 0.858]	0.251 [0.188, 0.368]	0.883 [0.861, 0.915]
Clinical_Only	Logistic	0.646 [0.572, 0.736]	0.278 [0.205, 0.391]	0.226 [0.212, 0.245]	0.708 [0.574, 0.793]	0.466 [0.330, 0.613]	0.752 [0.554, 0.869]	0.260 [0.185, 0.370]	0.887 [0.865, 0.913]
Clinical_Only	NN	0.608 [0.516, 0.696]	0.249 [0.179, 0.315]	0.194 [0.170, 0.220]	0.772 [0.688, 0.820]	0.263 [0.129, 0.444]	0.863 [0.742, 0.929]	0.260 [0.160, 0.379]	0.867 [0.850, 0.893]
Clinical_Only	Prevalence	0.500 [0.500, 0.500]	0.153 [0.153, 0.153]	0.129 [0.129, 0.129]	0.847 [0.847, 0.847]	0.000 [0.000, 0.000]	1.000 [1.000, 1.000]		0.847 [0.847, 0.847]
Clinical_Only	XGBoost	0.606 [0.519, 0.689]	0.250 [0.182, 0.337]	0.224 [0.198, 0.243]	0.165 [0.153, 0.283]	0.991 [0.903, 1.000]	0.016 [0.000, 0.174]	0.154 [0.153, 0.165]	0.939 [0.835, 1.000]
Clinical_Plus_SDH	BRF	0.597 [0.523, 0.670]	0.255 [0.185, 0.350]	0.229 [0.201, 0.242]	0.671 [0.519, 0.760]	0.434 [0.233, 0.645]	0.714 [0.497, 0.836]	0.218 [0.151, 0.267]	0.876 [0.847, 0.903]
Clinical_Plus_SDH	Dummy	0.500 [0.500, 0.500]	0.153 [0.153, 0.153]	0.129 [0.129, 0.129]	0.847 [0.847, 0.847]	0.000 [0.000, 0.000]	1.000 [1.000, 1.000]		0.847 [0.847, 0.847]
Clinical_Plus_SDH	ENet	0.623 [0.547, 0.699]	0.265 [0.199, 0.374]	0.228 [0.213, 0.246]	0.695 [0.555, 0.801]	0.444 [0.298, 0.638]	0.740 [0.532, 0.868]	0.241 [0.183, 0.356]	0.881 [0.860, 0.913]
Clinical_Plus_SDH	Logistic	0.635 [0.566, 0.725]	0.270 [0.199, 0.381]	0.227 [0.212, 0.247]	0.675 [0.536, 0.758]	0.487 [0.298, 0.727]	0.709 [0.513, 0.839]	0.236 [0.182, 0.309]	0.886 [0.861, 0.921]
Clinical_Plus_SDH	NN	0.595 [0.462, 0.671]	0.236 [0.148, 0.314]	0.197 [0.173, 0.243]	0.745 [0.336, 0.812]	0.279 [0.129, 0.823]	0.828 [0.248, 0.917]	0.248 [0.144, 0.365]	0.868 [0.846, 0.913]
Clinical_Plus_SDH	Prevalence	0.500 [0.500, 0.500]	0.153 [0.153, 0.153]	0.129 [0.129, 0.129]	0.847 [0.847, 0.847]	0.000 [0.000, 0.000]	1.000 [1.000, 1.000]		0.847 [0.847, 0.847]
Clinical_Plus_SDH	XGBoost	0.586 [0.496, 0.667]	0.236 [0.179, 0.349]	0.228 [0.194, 0.245]	0.153 [0.153, 0.153]	0.998 [1.000, 1.000]	0.001 [0.000, 0.000]	0.153 [0.153, 0.153]	0.727 [0.727, 0.727]

Note. Classification results are shown for the leakage-free clinical-only and clinical-plus-SDH predictor sets.

Supplementary Table S2. Full healthcare-utilization model performance.

Feature set	Model	MAE	RMSE	R2	Median AE
Clinical_Only	Dummy	5.279 [4.819, 5.779]	9.180 [7.529, 11.096]	-0.031 [-0.043, -0.021]	3.040 [3.000, 3.775]
Clinical_Only	RF	4.797 [4.187, 5.442]	7.369 [5.859, 8.731]	0.326 [0.060, 0.482]	3.369 [2.895, 3.921]
Clinical_Only	SVR	4.815 [4.236, 5.362]	7.766 [5.987, 8.996]	0.258 [0.147, 0.376]	3.179 [2.860, 3.604]
Clinical_Only	XGBoost	4.805 [4.196, 5.449]	7.425 [5.871, 8.955]	0.320 [0.172, 0.475]	3.352 [2.901, 3.791]
Clinical_Plus_SDH	Dummy	5.279 [4.819, 5.779]	9.180 [7.529, 11.096]	-0.031 [-0.043, -0.021]	3.040 [3.000, 3.775]
Clinical_Plus_SDH	RF	4.840 [4.213, 5.453]	7.425 [5.860, 8.776]	0.317 [0.127, 0.479]	3.426 [3.034, 3.947]
Clinical_Plus_SDH	SVR	4.838 [4.261, 5.375]	7.846 [6.092, 9.178]	0.244 [0.134, 0.340]	3.163 [2.883, 3.562]
Clinical_Plus_SDH	XGBoost	4.867 [4.325, 5.527]	7.575 [5.991, 9.116]	0.291 [0.068, 0.456]	3.389 [2.946, 3.876]

Note. Lower MAE, RMSE, and median absolute error indicate better performance; higher R2 indicates better explained variance.

Supplementary Table S3. Parsimonious clinical baseline performance for 90-day readmission.

Feature set	Model	AUROC	AUPRC	Brier	Accuracy	Sensitivity	Specificity
Parsimonious_Clinical	Dummy	0.500 [0.500, 0.500]	0.153 [0.153, 0.153]	0.129 [0.129, 0.129]	0.847 [0.847, 0.847]	0.000 [0.000, 0.000]	1.000 [1.000, 1.000]
Parsimonious_Clinical	Logistic	0.605 [0.556, 0.644]	0.242 [0.196, 0.294]	0.238 [0.231, 0.246]	0.662 [0.538, 0.742]	0.429 [0.323, 0.641]	0.704 [0.520, 0.817]
Parsimonious_Clinical	Prevalence	0.500 [0.500, 0.500]	0.153 [0.153, 0.153]	0.129 [0.129, 0.129]	0.847 [0.847, 0.847]	0.000 [0.000, 0.000]	1.000 [1.000, 1.000]

Note. This baseline used a small set of standard clinical variables to benchmark whether more complex models added measurable predictive signal.

Supplementary Table S4. Parsimonious clinical baseline performance for healthcare utilization.

Feature set	Model	MAE	RMSE	R2	Median AE
Parsimonious_Clinical	Dummy	5.302 [4.630, 5.759]	9.306 [7.062, 10.957]	-0.032 [-0.038, -0.025]	3.100 [3.000, 3.775]
Parsimonious_Clinical	SVR	4.916 [4.439, 5.317]	7.819 [6.170, 8.789]	0.264 [0.145, 0.407]	3.241 [3.032, 3.554]

Note. The parsimonious HU model was compared with a dummy model predicting central tendency.

Supplementary Table S5. Reconstructed cohort flow for complete-case analysis.

stage	n
Eligible encounters in cleaned workbook after age/date/known-readmission filters (reconstructed)	1050
Excluded for missing analysis value or unrecorded ASA class 0	35
Final analytic cohort (complete-case; matches Quest parquet n if reconstruction aligns)	1015
Quest analytic parquet n (authoritative)	1015

Note. The source cleaned workbook contained 1050 eligible encounters after age, date, and known-readmission filters; 35 were excluded because of missing analysis values or unrecorded ASA class.

Supplementary Table S6. Variables with missingness among eligible encounters before complete-case restriction.

Variable	Eligible n	Missing n	Missing %	Complete n	Note
anesthesia_duration_hours	1050	18	1.71	1032	
asa_class	1050	13	1.24	1037	ASA class 0 treated as unrecorded/missing
BMI	1050	6	0.57	1044	
LOS_days	1050	1	0.10	1049	
R_PL_THEME1	1050	1	0.10	1049	
R_PL_THEME2	1050	1	0.10	1049	
R_PL_THEME3	1050	1	0.10	1049	
R_PL_THEME4	1050	1	0.10	1049	
R_PL_THEMES	1050	1	0.10	1049	

Note. Variables not shown had no missingness among eligible encounters. ASA class 0 was treated as unrecorded/missing.

Supplementary Table S7. Full predictor variable list for final leakage-free models.

predictor_number	category	variable	included_in	timing
1	Demographic	age_at_procedure	Clinical_Only + Clinical_Plus_SDH	Index hospitalization / discharge-available
2	Demographic	BMI	Clinical_Only + Clinical_Plus_SDH	Index hospitalization / discharge-available
3	Demographic	asa_class	Clinical_Only + Clinical_Plus_SDH	Index hospitalization / discharge-available
4	Perioperative / index hospitalization	LOS_days	Clinical_Only + Clinical_Plus_SDH	Index hospitalization / discharge-available
5	Perioperative / index hospitalization	surgery_duration_hours	Clinical_Only + Clinical_Plus_SDH	Index hospitalization / discharge-available
6	Perioperative / index hospitalization	OR_duration_hours	Clinical_Only + Clinical_Plus_SDH	Index hospitalization / discharge-available
7	Perioperative / index hospitalization	anesthesia_duration_hours	Clinical_Only + Clinical_Plus_SDH	Index hospitalization / discharge-available
8	Perioperative / index hospitalization	surgery_year	Clinical_Only + Clinical_Plus_SDH	Index hospitalization / discharge-available
9	Preoperative medications	Opiods_60days_preop	Clinical_Only + Clinical_Plus_SDH	Index hospitalization / discharge-available
10	Preoperative medications	Benzodiazepine_60days_preop	Clinical_Only + Clinical_Plus_SDH	Index hospitalization / discharge-available
11	Preoperative medications	Neuromodulator_preop	Clinical_Only + Clinical_Plus_SDH	Index hospitalization / discharge-available
12	Clinical validation flags	validation_fmr_tobacco	Clinical_Only + Clinical_Plus_SDH	Index hospitalization / discharge-available
13	Clinical validation flags	validation_cur_tobacco	Clinical_Only + Clinical_Plus_SDH	Index hospitalization / discharge-available
14	Clinical validation flags	validation_myelopathy	Clinical_Only + Clinical_Plus_SDH	Index hospitalization / discharge-available
15	Elixhauser comorbidities	elix_aids_hiv	Clinical_Only + Clinical_Plus_SDH	Index hospitalization / discharge-available
16	Elixhauser comorbidities	elix_alcohol_abuse	Clinical_Only + Clinical_Plus_SDH	Index hospitalization / discharge-available
17	Elixhauser comorbidities	elix_blood_loss_anemia	Clinical_Only + Clinical_Plus_SDH	Index hospitalization / discharge-available
18	Elixhauser comorbidities	elix_cardiac_arrhythmia	Clinical_Only + Clinical_Plus_SDH	Index hospitalization / discharge-available
19	Elixhauser comorbidities	elix_chronic_pulmonary_disease	Clinical_Only + Clinical_Plus_SDH	Index hospitalization / discharge-available
20	Elixhauser comorbidities	elix_coagulopathy	Clinical_Only + Clinical_Plus_SDH	Index hospitalization / discharge-available
21	Elixhauser comorbidities	elix_congestive_heart_failure	Clinical_Only + Clinical_Plus_SDH	Index hospitalization / discharge-available
22	Elixhauser comorbidities	elix_deficiency_anemia	Clinical_Only + Clinical_Plus_SDH	Index hospitalization / discharge-available
23	Elixhauser comorbidities	elix_depression	Clinical_Only + Clinical_Plus_SDH	Index hospitalization / discharge-available
24	Elixhauser comorbidities	elix_diabetes_complicated	Clinical_Only + Clinical_Plus_SDH	Index hospitalization / discharge-available
25	Elixhauser comorbidities	elix_diabetes_uncomplicated	Clinical_Only + Clinical_Plus_SDH	Index hospitalization / discharge-available
26	Elixhauser comorbidities	elix_drug_abuse	Clinical_Only + Clinical_Plus_SDH	Index hospitalization / discharge-available
27	Elixhauser comorbidities	elix_fluid_and_electrolyte_disorders	Clinical_Only + Clinical_Plus_SDH	Index hospitalization / discharge-available
28	Elixhauser comorbidities	elix_hypertension_complicated	Clinical_Only + Clinical_Plus_SDH	Index hospitalization / discharge-available
29	Elixhauser comorbidities	elix_hypertension_uncomplicated	Clinical_Only + Clinical_Plus_SDH	Index hospitalization / discharge-available
30	Elixhauser comorbidities	elix_hypothyroidism	Clinical_Only + Clinical_Plus_SDH	Index hospitalization / discharge-available
31	Elixhauser comorbidities	elix_liver_disease	Clinical_Only + Clinical_Plus_SDH	Index hospitalization / discharge-available
32	Elixhauser comorbidities	elix_lymphoma	Clinical_Only + Clinical_Plus_SDH	Index hospitalization / discharge-available

predictor_number	category	variable	included_in	timing
33	Elixhauser comorbidities	elix_metastatic_cancer	Clinical_Only + Clinical_Plus_SDH	Index hospitalization / discharge-available
34	Elixhauser comorbidities	elix_obesity	Clinical_Only + Clinical_Plus_SDH	Index hospitalization / discharge-available
35	Elixhauser comorbidities	elix_other_neurological_disorder	Clinical_Only + Clinical_Plus_SDH	Index hospitalization / discharge-available
36	Elixhauser comorbidities	elix_paralysis	Clinical_Only + Clinical_Plus_SDH	Index hospitalization / discharge-available
37	Elixhauser comorbidities	elix_peptic_ulcer_disease_excluding_bleeding	Clinical_Only + Clinical_Plus_SDH	Index hospitalization / discharge-available
38	Elixhauser comorbidities	elix_peripheral vascular_disorder	Clinical_Only + Clinical_Plus_SDH	Index hospitalization / discharge-available
39	Elixhauser comorbidities	elix_psychoses	Clinical_Only + Clinical_Plus_SDH	Index hospitalization / discharge-available
40	Elixhauser comorbidities	elix_pulmonary_circulation_disorder	Clinical_Only + Clinical_Plus_SDH	Index hospitalization / discharge-available
41	Elixhauser comorbidities	elix_renal_failure	Clinical_Only + Clinical_Plus_SDH	Index hospitalization / discharge-available
42	Elixhauser comorbidities	elix_rheumatoid_arhritis	Clinical_Only + Clinical_Plus_SDH	Index hospitalization / discharge-available
43	Elixhauser comorbidities	elix_solid_tumor_wo_metastasis	Clinical_Only + Clinical_Plus_SDH	Index hospitalization / discharge-available
44	Elixhauser comorbidities	elix_valvular_disease	Clinical_Only + Clinical_Plus_SDH	Index hospitalization / discharge-available
45	Elixhauser comorbidities	elix_weight_loss	Clinical_Only + Clinical_Plus_SDH	Index hospitalization / discharge-available
46	Charlson comorbidities	charlson_aids_hiv	Clinical_Only + Clinical_Plus_SDH	Index hospitalization / discharge-available
47	Charlson comorbidities	charlson_cancer	Clinical_Only + Clinical_Plus_SDH	Index hospitalization / discharge-available
48	Charlson comorbidities	charlson_cerebrovascular_disease	Clinical_Only + Clinical_Plus_SDH	Index hospitalization / discharge-available
49	Charlson comorbidities	charlson_chronic_pulmonary_disease	Clinical_Only + Clinical_Plus_SDH	Index hospitalization / discharge-available
50	Charlson comorbidities	charlson_congestive_heart_failure	Clinical_Only + Clinical_Plus_SDH	Index hospitalization / discharge-available
51	Charlson comorbidities	charlson_connective_tissue_disease_rheumatic_disease	Clinical_Only + Clinical_Plus_SDH	Index hospitalization / discharge-available
52	Charlson comorbidities	charlson_dementia	Clinical_Only + Clinical_Plus_SDH	Index hospitalization / discharge-available
53	Charlson comorbidities	charlson_diabetes_w_complications	Clinical_Only + Clinical_Plus_SDH	Index hospitalization / discharge-available
54	Charlson comorbidities	charlson_diabetes_wo_complications	Clinical_Only + Clinical_Plus_SDH	Index hospitalization / discharge-available
55	Charlson comorbidities	charlson_metastitic_carcinoma	Clinical_Only + Clinical_Plus_SDH	Index hospitalization / discharge-available
56	Charlson comorbidities	charlson_mild_liver_disease	Clinical_Only + Clinical_Plus_SDH	Index hospitalization / discharge-available
57	Charlson comorbidities	charlson_moderate_or_sever_liver_disease	Clinical_Only + Clinical_Plus_SDH	Index hospitalization / discharge-available
58	Charlson comorbidities	charlson_myocardial_infarction	Clinical_Only + Clinical_Plus_SDH	Index hospitalization / discharge-available
59	Charlson comorbidities	charlson_paraplegia_and_hemiplegia	Clinical_Only + Clinical_Plus_SDH	Index hospitalization / discharge-available
60	Charlson comorbidities	charlson_peptic_ulcer_disease	Clinical_Only + Clinical_Plus_SDH	Index hospitalization / discharge-available
61	Charlson comorbidities	charlson_periphral_vascular_disease	Clinical_Only + Clinical_Plus_SDH	Index hospitalization / discharge-available
62	Charlson comorbidities	charlson_renal_disease	Clinical_Only + Clinical_Plus_SDH	Index hospitalization / discharge-available
63	mFI-5	mfi5_acute_hf	Clinical_Only + Clinical_Plus_SDH	Index hospitalization / discharge-available
64	mFI-5	mfi5_chf	Clinical_Only + Clinical_Plus_SDH	Index hospitalization / discharge-available

predictor_number	category	variable	included_in	timing
65	mFI-5	mfi5_copd	Clinical_Only + Clinical_Plus_SDH	Index hospitalization / discharge-available
66	mFI-5	mfi5_diabetes	Clinical_Only + Clinical_Plus_SDH	Index hospitalization / discharge-available
67	mFI-5	mfi5_func_status	Clinical_Only + Clinical_Plus_SDH	Index hospitalization / discharge-available
68	mFI-5	mfi5_hypertension	Clinical_Only + Clinical_Plus_SDH	Index hospitalization / discharge-available
69	mFI-11	mfi11_CVA_def	Clinical_Only + Clinical_Plus_SDH	Index hospitalization / discharge-available
70	mFI-11	mfi11_TIA_CVA_no_def	Clinical_Only + Clinical_Plus_SDH	Index hospitalization / discharge-available
71	mFI-11	mfi11_angina_prev_intervention	Clinical_Only + Clinical_Plus_SDH	Index hospitalization / discharge-available
72	mFI-11	mfi11_chf	Clinical_Only + Clinical_Plus_SDH	Index hospitalization / discharge-available
73	mFI-11	mfi11_copd	Clinical_Only + Clinical_Plus_SDH	Index hospitalization / discharge-available
74	mFI-11	mfi11_delirium_imp_sensor	Clinical_Only + Clinical_Plus_SDH	Index hospitalization / discharge-available
75	mFI-11	mfi11_diabetes	Clinical_Only + Clinical_Plus_SDH	Index hospitalization / discharge-available
76	mFI-11	mfi11_func_status	Clinical_Only + Clinical_Plus_SDH	Index hospitalization / discharge-available
77	mFI-11	mfi11_hypertension	Clinical_Only + Clinical_Plus_SDH	Index hospitalization / discharge-available
78	mFI-11	mfi11_mi	Clinical_Only + Clinical_Plus_SDH	Index hospitalization / discharge-available
79	mFI-11	mfi11_pvd_gangrene_rest_pain	Clinical_Only + Clinical_Plus_SDH	Index hospitalization / discharge-available
80	RAI	rai_CVA_def	Clinical_Only + Clinical_Plus_SDH	Index hospitalization / discharge-available
81	RAI	rai_TIA_CVA_no_def	Clinical_Only + Clinical_Plus_SDH	Index hospitalization / discharge-available
82	RAI	rai_cancer	Clinical_Only + Clinical_Plus_SDH	Index hospitalization / discharge-available
83	RAI	rai_chf	Clinical_Only + Clinical_Plus_SDH	Index hospitalization / discharge-available
84	RAI	rai_delirium_imparied	Clinical_Only + Clinical_Plus_SDH	Index hospitalization / discharge-available
85	RAI	rai_dyspnea	Clinical_Only + Clinical_Plus_SDH	Index hospitalization / discharge-available
86	RAI	rai_functional_status	Clinical_Only + Clinical_Plus_SDH	Index hospitalization / discharge-available
87	RAI	rai_renal_failure	Clinical_Only + Clinical_Plus_SDH	Index hospitalization / discharge-available
88	RAI	rai_weight_loss	Clinical_Only + Clinical_Plus_SDH	Index hospitalization / discharge-available
89	Census-tract SVI themes (SDH)	R_PL_THEME1	Clinical_Plus_SDH only	Census-tract SVI (area-level; address at surgery)
90	Census-tract SVI themes (SDH)	R_PL_THEME2	Clinical_Plus_SDH only	Census-tract SVI (area-level; address at surgery)
91	Census-tract SVI themes (SDH)	R_PL_THEME3	Clinical_Plus_SDH only	Census-tract SVI (area-level; address at surgery)
92	Census-tract SVI themes (SDH)	R_PL_THEME4	Clinical_Plus_SDH only	Census-tract SVI (area-level; address at surgery)
93	Census-tract SVI themes (SDH)	R_PL_THEMES	Clinical_Plus_SDH only	Census-tract SVI (area-level; address at surgery)

Note. The clinical-only candidate set contained 88 predictors. The clinical-plus-SDH set added five SVI measures for 93 total predictors. No post-discharge utilization components, postoperative complications, or readmission variables were used as predictors.

Supplementary Table S8. Paired incremental comparison of clinical-plus-SDH versus clinical-only models.

Model	Metric	Delta definition	Repetitions	Mean delta	SD	2.5th pct	97.5th pct	Fraction plus model better	Wilcoxon p
Logistic	AUC	Clinical_Plus_SDH - Clinical_Only	50	-0.011	0.011	-0.033	0.006	0.080	4.72e-08
Logistic	AUPRC	Clinical_Plus_SDH - Clinical_Only	50	-0.009	0.010	-0.029	0.006	0.080	5.90e-10
Logistic	Brier	Clinical_Plus_SDH - Clinical_Only	50	9.80e-04	0.002	-0.002	0.004	0.260	1.52e-05
Logistic	ECE	Clinical_Plus_SDH - Clinical_Only	50	-8.11e-04	0.006	-0.009	0.011	0.600	0.0805
Logistic	AUC	Clinical_Plus_Theme1 - Clinical_Only	10	-0.004	0.005	-0.012	0.002	0.200	0.0488
Logistic	AUPRC	Clinical_Plus_Theme1 - Clinical_Only	10	-0.003	0.010	-0.022	0.004	0.600	0.6953
Logistic	Brier	Clinical_Plus_Theme1 - Clinical_Only	10	3.80e-04	8.10e-04	-7.37e-04	0.001	0.400	0.3223
Logistic	ECE	Clinical_Plus_Theme1 - Clinical_Only	10	3.22e-04	0.001	-0.001	0.003	0.600	1.0000
SVR	MAE	Clinical_Plus_SDH - Clinical_Only	50	0.023	0.058	-0.069	0.174	0.340	0.0022
SVR	RMSE	Clinical_Plus_SDH - Clinical_Only	50	0.080	0.164	-0.131	0.531	0.260	2.21e-04
SVR	R2	Clinical_Plus_SDH - Clinical_Only	50	-0.015	0.029	-0.086	0.026	0.260	2.86e-04
SVR	MAE	Clinical_Plus_Theme34 - Clinical_Only	10	0.014	0.026	-0.020	0.063	0.300	0.1309
SVR	RMSE	Clinical_Plus_Theme34 - Clinical_Only	10	0.091	0.115	-0.028	0.258	0.300	0.0645
SVR	R2	Clinical_Plus_Theme34 - Clinical_Only	10	-0.016	0.021	-0.047	0.007	0.300	0.0840

Note. For AUROC, AUPRC, and R2, positive deltas favor the plus-feature model. For Brier, ECE, MAE, and RMSE, negative deltas favor the plus-feature model.

Supplementary Table S9. Post-hoc recalibration sensitivity for the clinical-plus-SDH logistic model.

Method	Repetitions	Brier	ECE	Calibration slope	Calibration-in-the-large	Mean predicted risk	Observed prevalence
isotonic	50	0.127	0.037	0.563	0.003	0.156	0.153
platt	50	0.126	0.033	0.976	0.003	0.156	0.153
raw	50	0.227	0.313	0.926	0.311	0.464	0.153

Note. Raw class-weighted predictions overestimated absolute readmission risk. Platt and isotonic recalibration were post-hoc sensitivity analyses performed within the repeated-split workflow.

Supplementary Table S10. Sensitivity analyses for restricted SVI and PCA specifications.

Sensitivity analysis	Outcome	Model	Metric	Delta definition	Repetitions	Mean delta	2.5th pct	97.5th pct	Fraction plus model better	Wilcoxon p
restricted_svi	readmission	Logistic	AUC	Clinical_Plus_Theme1 - Clinical_Only	10	-0.004	-0.012	0.002	0.200	0.0488
restricted_svi	readmission	Logistic	AUPRC	Clinical_Plus_Theme1 - Clinical_Only	10	-0.003	-0.022	0.004	0.600	0.6953
restricted_svi	readmission	Logistic	Brier	Clinical_Plus_Theme1 - Clinical_Only	10	3.80e-04	-7.37e-04	0.001	0.400	0.3223
restricted_svi	readmission	Logistic	ECE	Clinical_Plus_Theme1 - Clinical_Only	10	3.22e-04	-0.001	0.003	0.600	1.0000
pca	readmission	Logistic	AUC	Clinical_Plus_SDH_PCA - Clinical_Only_PCA	10	-0.013	-0.034	-0.004	0.000	0.0020
pca	readmission	Logistic	AUPRC	Clinical_Plus_SDH_PCA - Clinical_Only_PCA	10	-0.009	-0.036	0.003	0.200	0.0195
pca	readmission	Logistic	Brier	Clinical_Plus_SDH_PCA - Clinical_Only_PCA	10	0.002	-2.38e-04	0.005	0.100	0.0039
pca	readmission	Logistic	ECE	Clinical_Plus_SDH_PCA - Clinical_Only_PCA	10	0.001	-0.002	0.010	0.400	0.5566
restricted_svi	HU	SVR	MAE	Clinical_Plus_Theme34 - Clinical_Only	10	0.014	-0.020	0.063	0.300	0.1309
restricted_svi	HU	SVR	RMSE	Clinical_Plus_Theme34 - Clinical_Only	10	0.091	-0.028	0.258	0.300	0.0645
restricted_svi	HU	SVR	R2	Clinical_Plus_Theme34 - Clinical_Only	10	-0.016	-0.047	0.007	0.300	0.0840
pca	HU	SVR	MAE	Clinical_Plus_SDH_PCA - Clinical_Only_PCA	10	0.002	-0.069	0.065	0.500	0.9219
pca	HU	SVR	RMSE	Clinical_Plus_SDH_PCA - Clinical_Only_PCA	10	-1.86e-05	-0.120	0.109	0.500	0.9219
pca	HU	SVR	R2	Clinical_Plus_SDH_PCA - Clinical_Only_PCA	10	7.64e-04	-0.017	0.022	0.500	0.8457

Note. Restricted SVI models used Theme 1 for readmission and Themes 3/4 for HU based on SHAP attribution. PCA analyses summarized correlated SVI measures.

Supplementary Table S11. SVI theme ranks from SHAP attribution.

Analysis	Overall rank	SVI feature	Mean absolute SHAP	SD absolute SHAP	Median rank	Rank 2.5th pct	Rank 97.5th pct	Fraction top 10	Fraction top 20	Repetitions
readmission_Logistic	7	R_PL_THEME1	0.054	0.040	8.0	3.225	23.550	0.800	0.920	50
readmission_Logistic	12	R_PL_THEME3	0.032	0.025	12.0	5.225	62.550	0.400	0.740	50
readmission_Logistic	14	R_PL_THEMES	0.027	0.035	14.0	7.000	79.200	0.180	0.740	50
readmission_Logistic	16	R_PL_THEME2	0.024	0.026	16.0	7.000	70.200	0.280	0.560	50
readmission_Logistic	19	R_PL_THEME4	0.023	0.019	16.0	7.000	75.100	0.320	0.600	50
HU_SVR	6	R_PL_THEME3	0.221	0.059	7.0	5.225	10.325	0.900	1.000	10
HU_SVR	7	R_PL_THEME4	0.166	0.117	9.0	5.225	47.225	0.700	0.900	10
HU_SVR	8	R_PL_THEMES	0.151	0.106	12.0	5.450	21.875	0.400	0.900	10
HU_SVR	16	R_PL_THEME1	0.081	0.059	18.5	7.000	80.250	0.200	0.600	10
HU_SVR	51	R_PL_THEME2	0.031	0.031	50.0	19.500	84.650	0.000	0.100	10

Note. Ranks summarize where each SVI feature appeared among all predictors across repeated analyses. Mean absolute SHAP values represent average absolute contribution magnitude to the model output.

Supplementary Table S12. Hyperparameter search spaces and tuning protocol.

Outcome/model type	Model	Tuned hyperparameters and search space	Optimization metric	Search method
90-day readmission classification	Logistic regression	C: Real(0.01-100.0; log-uniform)	roc_auc	Bayesian optimization (scikit-optimize BayesSearchCV); 25 iterations; 5-fold (stratified for classification) inner CV
90-day readmission classification	Elastic-net logistic regression	C: Real(0.01-100.0; log-uniform); l1_ratio: Real(0.0-1.0; uniform)	roc_auc	Bayesian optimization (scikit-optimize BayesSearchCV); 25 iterations; 5-fold (stratified for classification) inner CV
90-day readmission classification	Balanced random forest	n_estimators: Integer(50-250); max_depth: Integer(1-25); min_samples_split: Integer(2-10); min_samples_leaf: Integer(1-5); max_features: Real(0.3-1.0; uniform)	roc_auc	Bayesian optimization (scikit-optimize BayesSearchCV); 25 iterations; 5-fold (stratified for classification) inner CV
90-day readmission classification	XGBoost	n_estimators: Integer(50-250); max_depth: Integer(2-10); subsample: Real(0.5-1.0; uniform); learning_rate: Real(0.01-0.5; log-uniform); colsample_bytree: Real(0.5-1.0; uniform); min_child_weight: Integer(1-10); gamma: Real(0.0-1.0; uniform); reg_lambda: Real(0.01-100.0; log-uniform); reg_alpha: Real(0.01-100.0; log-uniform)	roc_auc	Bayesian optimization (scikit-optimize BayesSearchCV); 25 iterations; 5-fold (stratified for classification) inner CV
Healthcare-utilization regression	Support vector regression	kernel: Categorical(rbf, linear); C: Real(0.01-100.0; log-uniform); gamma: Real(0.0001-1.0; log-uniform); epsilon: Real(0.01-1.0; uniform)	neg_mean_absolute_error	Bayesian optimization (scikit-optimize BayesSearchCV); 25 iterations; 5-fold (stratified for classification) inner CV
Healthcare-utilization regression	Random forest regression	n_estimators: Integer(50-250); max_depth: Integer(1-25); min_samples_split: Integer(2-10); min_samples_leaf: Integer(1-5); max_features: Real(0.3-1.0; uniform)	neg_mean_absolute_error	Bayesian optimization (scikit-optimize BayesSearchCV); 25 iterations; 5-fold (stratified for classification) inner CV
Healthcare-utilization regression	XGBoost	n_estimators: Integer(50-250); max_depth: Integer(2-10); subsample: Real(0.5-1.0; uniform); learning_rate: Real(0.01-0.5; log-uniform); colsample_bytree: Real(0.5-1.0; uniform); min_child_weight: Integer(1-10); gamma: Real(0.0-1.0; uniform); reg_lambda: Real(0.01-100.0; log-uniform); reg_alpha: Real(0.01-100.0; log-uniform)	neg_mean_absolute_error	Bayesian optimization (scikit-optimize BayesSearchCV); 25 iterations; 5-fold (stratified for classification) inner CV
90-day readmission classification	Neural network	fixed MLP 64-32, dropout 0.3, Adam, early stopping patience 10, val_split 0.15	Fixed architecture; early stopping on validation split	No Bayesian search for neural-network architecture
90-day readmission classification	Dummy/no-skill reference	None	No-skill comparator	No hyperparameter search
90-day readmission classification	Prevalence-only reference	None; predicts training-set prevalence	Prevalence-only calibration reference	No hyperparameter search
Healthcare-utilization regression	Dummy/no-skill reference	None; predicts training-set median	No-skill comparator	No hyperparameter search

Note. Hyperparameters were tuned only within training folds. The search spaces summarize the pre-specified tuning grid or distribution for each model family.

Supplementary Table S13. TRIPOD+AI checklist for the revised PCDF prediction-model manuscript.

Item	Topic	TRIPOD+AI reporting element	Status	Manuscript location	Comment
1	Title	Identify study as prediction model work, with target population and outcomes.	Partially reported	Title page	Title identifies predictive analysis in PCDF, but does not explicitly name both outcomes or state multivariable prediction model evaluation.
2	Abstract	Summarize healthcare context, data, model, validation, results, and interpretation.	Reported	Abstract	Abstract reports retrospective cohort, PCDF population, outcomes, models, repeated splits, key performance, calibration concern, and exploratory interpretation.
3a	Background	Explain healthcare context and rationale for model development/evaluation.	Reported	Introduction	PCDF risk, postoperative HU, SDH rationale, and ML/XAI motivation are described.
3b	Background	Describe target population, intended purpose, care-pathway context, and intended users.	Partially reported	Introduction; Methods 2.1; Conclusion	Target population and discharge-time exploratory purpose are described; intended users are implied as perioperative teams but not named explicitly.
3c	Background	Describe relevant health inequalities across sociodemographic groups.	Reported	Introduction; Discussion	Prior SDH-related disparities in spine surgery and perioperative outcomes are summarized.
4	Objectives	State whether the study develops, evaluates, validates, or compares prediction models.	Reported	End of Introduction	Objective states evaluation of whether SVI adds incremental predictive value beyond clinical variables for readmission and HU.
5a	Data	Describe data sources and rationale/representativeness.	Partially reported	Methods 2.1; Limitations	Single-institution EDW source and rationale are reported; representativeness is addressed mainly as a limitation.
5b	Data	Specify dates of data collection, accrual, and follow-up horizon.	Reported	Methods 2.1	Cohort dates are 2003-2023, and outcomes are assessed within 90 days after discharge.
6a	Participants	Describe study setting, number/location of centers.	Reported	Methods 2.1; Author affiliations	Single-institution academic health system is reported.
6b	Participants	Describe eligibility criteria.	Reported	Methods 2.1	Adult single- or multilevel PCDF for degenerative disease; exclusions for malignancy, trauma, infection, and incomplete analysis data.
6c	Participants	Describe treatments received and handling in model analyses, if relevant.	Partially reported	Methods 2.1; Table 2	Index PCDF and multilevel status are described, but detailed operative treatment granularity is limited.
7	Data preparation	Describe preprocessing and quality checks, including across sociodemographic groups if assessed.	Partially reported	Methods 2.1-2.2; Supplementary Data	Complete-case analysis, standardization, geocoding/SVI linkage, and training-only preprocessing are described; quality checks across sociodemographic groups are not described.
8a	Outcome	Define predicted outcomes, time horizon, assessment method, rationale, and consistency.	Reported	Methods 2.1; Introduction	90-day readmission and HU score are defined; HU rationale is described.
8b	Outcome	If subjective outcome assessment was used, describe assessor qualifications/demographics.	Partially reported	Methods 2.1	Readmission relatedness involves clinical interpretation, but assessor qualifications/demographics are not described.
8c	Outcome	Report blinding of outcome assessment, if applicable.	Not reported	Not identified	No blinding procedure is described.
9a	Predictors	Describe initial predictor selection and any preselection.	Partially reported	Methods 2.1; Table 1a	Predictor domains and full variables are listed; rationale for each initial predictor source/preselection is only broadly stated.
9b	Predictors	Define predictors, timing, measurement, and blinding if relevant.	Reported	Methods 2.1; Table 1a	Predictor domains, discharge-time availability, medication timing, SVI source, and leakage exclusions are described.
9c	Predictors	If predictor measurement required subjective interpretation, describe assessors.	Partially reported	Methods 2.1	Chart-address abstraction/geocoding are described, but abstractor qualifications/demographics are not.
10	Sample size	Explain study-size derivation and justify adequacy, including calculations if used.	Partially reported	Methods 2.1; Results; Limitations	Cohort derivation and event count are reported; no formal sample-size calculation is provided.
11	Missing data	Describe missing-data handling and reasons for omitted data.	Reported	Methods 2.1; Supplementary Data	Complete-case analysis, 35 exclusions, ASA/unrecorded handling, and no imputation are described.
12a	Analytical methods	Describe data partitioning for model development/evaluation.	Reported	Methods 2.2	Fifty repeated 80/20 train-test splits are described, with stratification/quintile splitting as appropriate.
12b	Analytical methods	Describe predictor handling, transformations, scaling, or standardization.	Reported	Methods 2.2	Continuous variables were standardized and binary variables passed through unchanged.
12c	Analytical methods	Specify model types, modeling steps, tuning, and internal validation.	Reported	Methods 2.2; Supplementary Data	Model families, Bayesian hyperparameter search, inner 5-fold CV, threshold selection, and internal repeated-split evaluation are described.
12d	Analytical methods	Describe heterogeneity across clusters if applicable.	Not applicable	Methods 2.1; Limitations	Single-center cohort; no cluster-level performance analysis was performed.
12e	Analytical methods	Specify performance measures and plots used to evaluate models.	Reported	Methods 2.2; Results; Figures; Supplementary Data	AUROC, AUPRC, Brier, calibration, threshold metrics, MAE/RMSE/R2, SHAP, decision-curve analysis, and sensitivity analyses are reported.

Item	Topic	TRIPOD+AI reporting element	Status	Manuscript location	Comment
12f	Analytical methods	Describe model updating/recalibration arising from evaluation.	Reported	Methods 2.2; Results	Post-hoc nested Platt and isotonic recalibration sensitivity analyses are described.
12g	Analytical methods	Describe how model predictions were calculated for evaluation.	Partially reported	Methods 2.2	Python packages and model classes are listed, but deployable formulas/model objects/code are not provided.
13	Class imbalance	Describe class-imbalance handling and recalibration if used.	Reported	Methods 2.2; Results	Balanced class weights, XGBoost scale-pos-weight, and post-hoc recalibration sensitivity are described.
14	Fairness	Describe fairness approaches and rationale.	Partially reported	Introduction; Methods 2.1; Discussion	The study evaluates SVI/SDH attribution and incremental value, but formal fairness metrics or mitigation procedures are not reported.
15	Model output	Specify prediction output and any classification thresholds.	Reported	Methods 2.2; Results	Readmission models output risk/classification at a training-derived F1 threshold; HU models output continuous HU predictions.
16	Training versus evaluation	Identify differences between development and evaluation data.	Not applicable	Methods 2.2	Internal repeated train-test splits were drawn from the same cohort; no separate external evaluation dataset was used.
17	Ethical approval	Report IRB/ethics approval and consent/waiver status.	Reported	Institutional Review Board Statement; Informed Consent Statement	Northwestern IRB protocol STU00214424 and approval history are reported; informed consent is listed as not applicable.
18a	Funding	Report funding source and funder role.	Reported	Funding	No external funding is reported.
18b	Conflicts of interest	Disclose conflicts and financial interests.	Reported	Conflicts of Interest	No conflicts or financial disclosures are reported.
18c	Protocol	State whether protocol exists and where it is accessible.	Not reported	Not identified	No study protocol statement was identified.
18d	Registration	Provide study registration information or state not registered.	Not reported	Not identified	No registration statement was identified.
18e	Data sharing	Describe study-data availability.	Reported	Data Availability Statement	Raw data are stated to be available from authors on request.
18f	Code sharing	Describe analytic-code availability.	Not reported	Not identified	Analytic code availability is not stated.
19	Patient/public involvement	Describe patient/public involvement or state none.	Not reported	Not identified	No patient or public involvement statement was identified.
20a	Participants/results	Describe participant flow, outcomes, and follow-up summary.	Partially reported	Methods 2.1; Results; Table 2	Eligible and final cohort sizes, readmission events, and 90-day horizon are reported; no flow diagram is included.
20b	Participants/results	Report participant characteristics, key predictors, treatments, outcomes, missingness, and demographic differences.	Reported	Results; Tables 2 and 4; Supplementary Data	Overall demographics and stratified descriptive comparisons are reported; missingness details are in supplementary tables.
20c	Participants/results	For external evaluation, compare evaluation data with development data.	Not applicable	Methods 2.2	No external evaluation dataset was used.
21	Model development	Report participants and outcome events in each analysis.	Partially reported	Results; Methods 2.2	Total analytic cohort and readmission events are reported; repeated split sizes are implied by 80/20 design.
22	Model specification	Provide full model details sufficient for third-party prediction or implementation.	Partially reported	Methods 2.2; Table 1a; Supplementary Data	Predictor list, model classes, and hyperparameter search are available, but fitted model objects/formulas or code are not provided.
23a	Model performance	Report model performance with intervals and key subgroup analyses if relevant.	Partially reported	Results; Table 3; Supplementary Data	Performance estimates and split-based intervals/SDs are reported; subgroup performance by sociodemographic group is not reported.
23b	Model performance	Report cluster heterogeneity if examined.	Not applicable	Not applicable	No multi-center or cluster-level heterogeneity analysis was performed.
24	Model updating	Report recalibration/model updating results.	Reported	Methods 2.2; Results; Supplementary Data	Platt/isotonic recalibration sensitivity results are reported.
25	Discussion	Interpret results, including fairness, objectives, and prior studies.	Reported	Discussion	Discussion interprets modest performance, lack of SVI incremental value, SHAP attribution, and equity context.
26	Limitations	Discuss limitations, bias, uncertainty, and generalizability.	Reported	Limitations	Single-center design, complete-case analysis, event-to-predictor ratio, SVI proxy limitations, calibration, and need for external validation are discussed.
27a	Usability	Describe handling of poor or unavailable input data for implementation.	Not applicable	Conclusion; Limitations	Models are presented as exploratory and not clinically deployable, so implementation handling is not specified.
27b	Usability	Specify user interaction/expertise required for model use.	Not applicable	Conclusion	No clinical deployment workflow is proposed.
27c	Usability	Discuss next research steps for applicability and generalizability.	Reported	Discussion; Conclusion	Future external validation, calibration, and SDH-informed intervention studies are described.

Abbreviations: ACDF = anterior cervical discectomy and fusion; AUPRC = area under the precision-recall curve; AUROC = area under the receiver operating characteristic curve; CV = cross-validation; EDW = electronic data warehouse; HU = healthcare utilization; IRB = institutional review board; ML = machine learning; PCDF = posterior cervical decompression and fusion; SDH = social determinants of health; SHAP = Shapley Additive Explanations; SVI = Social Vulnerability Index; XAI = explainable artificial intelligence.

Source: TRIPOD+AI reporting guideline for studies developing or evaluating artificial intelligence prediction models.

Supplementary Table S14. Healthcare-utilization domain definitions used for domain-specific sensitivity analyses.

HU domain	Component variable included
Outpatient visits	Number_of_neurosurgery_visits
Outpatient visits	Number_of_orthopaedic_visits
Outpatient visits	Number_of_primary_care_visits
Outpatient visits	Number_of_urgent_care_visits
Outpatient visits	Number_of_PT_encounters
Acute care	Number_of_ED
Acute care	Number_of_hospitalizations
Acute care	Number_of_ICU
Referrals	Number_of_PT_referrals
Referrals	Number_of_other_referrals
Imaging and procedures	Number_of_imaging_orders
Imaging and procedures	Number_of_cardiac_procedures
Imaging and procedures	Number_of_venous_duplex_procedures
Imaging and procedures	Number_of_drainage_aspirations

Imaging and procedures	Number_of_IVC_filter
Imaging and procedures	Number_of_invasive_procedures
Imaging and procedures	Number_of_pulmonary_procedures
Imaging and procedures	Number_of_EMG_NCS_procedures

HU = healthcare utilization; ED = emergency department; ICU = intensive care unit; PT = physical therapy; IVC = inferior vena cava; EMG = electromyography; NCS = nerve conduction study. Domain scores were calculated as unweighted sums of the listed HU components and used as exploratory sensitivity outcomes. The total HU score remained the primary HU outcome. No item-level clinical weights were applied because no prespecified weighting scheme was available.
